# BI-2536 Promotes Neuroblastoma Cell Death via Minichromosome Maintenance Complex Components 2 and 10

**DOI:** 10.3390/ph15010037

**Published:** 2021-12-28

**Authors:** Chiao-Hui Hsieh, Hsiang-Ning Yeh, Chen-Tsung Huang, Wei-Hsuan Wang, Wen-Ming Hsu, Hsuan-Cheng Huang, Hsueh-Fen Juan

**Affiliations:** 1 Department of Life Science, National Taiwan University, Taipei 10617, Taiwan; ab032808@hotmail.com.tw (C.-H.H.); shining.yeh36@yahoo.com.tw (H.-N.Y.); 2 Graduate Institute of Biomedical Electronics and Bioinformatics, National Taiwan University, Taipei 10617, Taiwan; b94901092@ntu.edu.tw; 3 Genome and Systems Biology Degree Program, National Taiwan University and Academia Sinica, Taipei 11529, Taiwan; weihsuan15@gmail.com; 4 Department of Surgery, National Taiwan University Hospital and National Taiwan University College of Medicine, Taipei 10017, Taiwan; billwmhsu@gmail.com; 5 Institute of Biomedical Informatics, Center for Systems and Synthetic Biology, National Yang Ming Chiao Tung University, Taipei 11221, Taiwan; 6 Center for Computational and Systems Biology, National Taiwan University, Taipei 10617, Taiwan

**Keywords:** minichromosome maintenance complex 2 and 10, BI-2536, neuroblastoma, mitochondria fusion, cell death

## Abstract

DNA replication is initiated with the recognition of the starting point of multiple replication forks by the origin recognition complex and activation of the minichromosome maintenance complex 10 (MCM10). Subsequently, DNA helicase, consisting of the MCM protein subunits MCM2-7, unwinds double-stranded DNA and DNA synthesis begins. In previous studies, replication factors have been used as clinical targets in cancer therapy. The results showed that MCM2 could be a proliferation marker for numerous types of malignant cancer. We analyzed samples obtained from patients with neuroblastoma, revealing that higher levels of MCM2 and MCM10 mRNA were associated with poor survival rate. Furthermore, we combined the results of the perturbation-induced reversal effects on the expression levels of MCM2 and MCM10 and the sensitivity correlation between perturbations and MCM2 and MCM10 from the Cancer Therapeutics Response Portal database. Small molecule BI-2536, a polo-like kinase 1 (PLK-1) inhibitor, is a candidate for the inhibition of MCM2 and MCM10 expression. To test this hypothesis, we treated neuroblastoma cells with BI-2536. The results showed that the drug decreased cell viability and reduced the expression levels of MCM2 and MCM10. Functional analysis further revealed enrichments of gene sets involved in mitochondria, cell cycle, and DNA replication for BI-2536-perturbed transcriptome. We used cellular assays to demonstrate that BI-2536 promoted mitochondria fusion, G2/M arrest, and apoptosis. In summary, our findings provide a new strategy for neuroblastoma therapy with BI-2536.

## 1. Introduction

Neuroblastoma is the third most common type of cancer in children, with an overall survival rate <40%. The disease affects 10.2 per million children aged <15 years [1]. Common symptoms include a lump or swelling in the neck, chest, or abdomen, fever, limping, fatigue, bruising, or bone pain (in case of bone metastasis) [2,3]. Although patients with localized disease can be cured through surgery, the long-term survival rate of children aged >1.5 years treated with multimodal therapy remains poor. Surgery, radiation therapy, chemotherapy, immunotherapy, stem cell transplantation, and the differentiation agent isotretinoin are modalities commonly used in neuroblastoma therapy [4,5]. Despite the large amount of research conducted on cancer drug development, there is currently no effective drug against neuroblastoma without adverse effects.

Unregulated DNA replication usually occurs in tumor cells; thus, inhibition of this replication may be an effective therapeutic intervention for cancer therapy [6,7]. The minichromosome maintenance complex (MCM) is involved in DNA replication, and plays a crucial role in the cell cycle and proliferation, particularly in malignant cells. DNA helicase is composed of a MCM2-7 hetero-hexamer [8,9] and unwinds double-stranded DNA (dsDNA) in the process of DNA replication. MCM10 also interacts with MCM2 and MCM6; this is essential for the recruitment of MCM2–7 to initiate DNA replication [10]. Additionally, MCM is a proliferation marker in numerous types of cancer (e.g., breast, lung, and ovarian) [11,12,13,14]. Previous reports have shown that both upregulation and downregulation of MCM are associated with cancer progression [11,13,14]. In addition, our previous study showed that some non-oncogene dependencies may serve as better therapeutic targets for high-risk neuroblastoma. MCM10, MCM2, MCM3, and MCM6 are among the high-risk signature correlating with poor prognosis in both MYCN-amplified and MYCN-non-amplified neuroblastoma [15].

The aim of this study was to identify potential compounds that may simultaneously inhibit MCM2 and MCM10 to repress cell genome duplication and division leading to cell death. Furthermore, we investigated relationships between small molecules and cancer genome alternations to predict substitutional drugs for neuroblastoma, using data obtained from the Library of Integrated Network-based Cellular Signatures (LINCS) and the Cancer Therapeutics Response Portal database [16]. We validated the cytotoxicity and mechanisms of BI-2536 in neuroblastoma, shedding new light on a promising compound to effectively treat high-risk neuroblastoma.

## 2. Results

### 2.1. Role of MCM2 and MCM10 in Neuroblastoma

**Figure 1:**
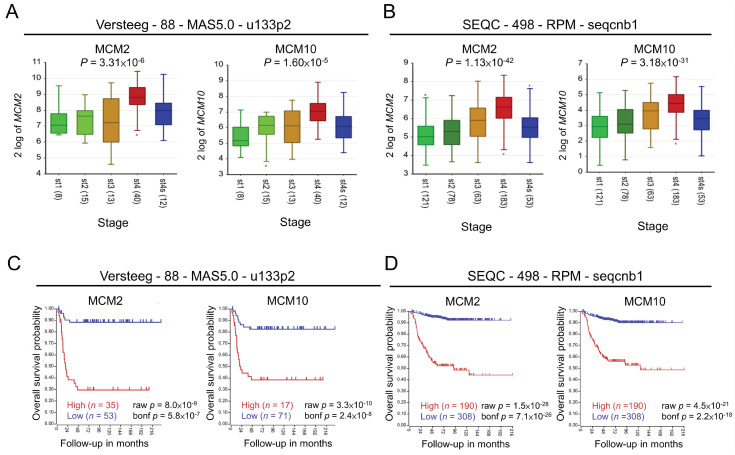
Expression of MCM complex genes across INSS stages and their association with survival probability: (**A**,**B**) Boxplots of MCM2 and MCM10 expression with respective to different INSS stages from two data sets. The expression of MCM2 and MCM10 was relatively low in patients with stage 4S neuroblastoma, in accordance with better prognosis associated with this stage of disease. The *x*-axis represents the INSS stage with patient numbers shown in parentheses. *p*-values were computed using one-way ANOVA. The “x value” is indicated as the outlier. (**C**,**D**) Kaplan–Meier curve analysis of patients with neuroblastoma stratified by MCM2 or MCM10 expression (*n* = 88 and *n* = 498 samples in each data set). High and low expression of a given gene was shown as red and blue, respectively. INSS, International Neuroblastoma Staging System. *p*-values were computed using log-rank test (raw *p*) and then corrected with the Bonferroni method (bond *p*).

We first examined whether the expression of MCM genes correlated with the tumor stage based on the International Neuroblastoma Staging System (INSS) for neuroblastoma. We found that expression of several MCM genes, except for MCM9, positively correlated with advanced disease stage and was relatively lower in stage 4S neuroblastoma, which usually exhibits better prognosis compared with other stages (Figure 1A,B, Figures S1 and S2, Supplementary Materials). Kaplan–Meier curve analysis also showed that high expression of MCM genes, except for MCM9, were associated with poor survival in neuroblastoma (Figure 1C,D, Figures S3 and S4, Supplementary Materials), even in patients with MYCN-non-amplified tumor (Figure S5, Supplementary Materials). The survival rates were not significantly changed in patients with high or low expression of MCM2 or MCM10 with MYCN-amplified neuroblastoma, suggesting that MYCN amplification is more dominant than MCM expression in determining poor prognosis in this patient subgroup (Figure S5). Because MYCN has been shown to drive neuroblastoma pathogenesis, we used the high-risk gene signature (high-risk MYCN non-amplified subgroup) to predict potential target [15]. Analysis of the expression of MCM2 (Figure S6A, Supplementary Materials) and MCM10 (Figure S6B) in 35 types of cancer was performed using the Affymetrix array HG-U133_Plus_2 platform from the GENT2 database (http://gent2.appex.kr/gent2/; (accessed on 24 July 2021)). In most types of cancer (except for adipose, bone, and tooth cancers), the expression of MCM2 and MCM10 was higher in cancerous tissues than non-cancerous tissues. This evidence supports a potential causal relationship between MCM genes and the development of neuroblastoma. Based on these findings, we selected MCM2 and MCM10 as ideal targets for functional evaluation in our study.

### 2.2. Knockdown of MCM2 and MCM10 Suppressed Neuroblastoma Cell Growth

**Figure 2:**
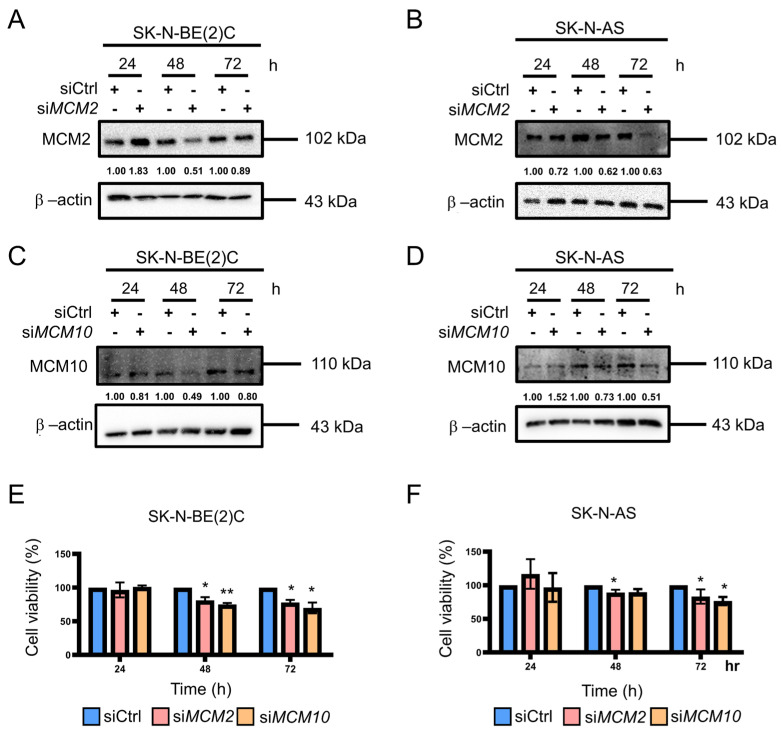
Knockdown of MCM2 and MCM10 reduced neuroblastoma cell proliferation: (**A**–**D**) Knockdown efficiency of MCM2 and MCM10 in SK-N-BE (2) C (**A**,**C**) and SK-N-AS (**B**,**D**) 24, 48, or 72 h post-transfection was confirmed with Western blot. (**E**,**F**) Cell viability in MCM2 or MCM10 silenced neuroblastoma cells. * *p* < 0.05, ** *p* < 0.01 from two-tailed unpair student *t*-test to determine whether the mean value of a given group is equal to the corresponding siCtrl group.

In order to investigate the importance of MCM2 and MCM10 in neuroblastoma, siRNA was used for the transient knockdown of the target genes in the MYCN-amplified cell line SK-N-BE(2)C and the MYCN-nonamplified cell line SK-N-AS. Western blotting was used to determine the knockdown efficiency (Figure 2A–D and Figure S7, Supplementary Materials). Both MCM2 and MCM10 were knocked down at 48 and 72 h post transfection. Cell viability was measured at 24, 48, and 72 h post transfection by cell count. In SK-N-BE(2)C cells, the proliferation rate of MCM2 and MCM10 silenced cells decreased by 17% and 14% for 48 h and by 20% and 35% for 72 h, respectively, compared with that observed in DMSO group (Figure 2E). Similarly, in SK-N-AS cells, the proliferation rate of MCM2 and MCM10 silenced cells decreased by 8% and 8% for 48 h and by 18% and 27% for 72 h, respectively (Figure 2F). These data indicate that MCM2 and MCM10 contribute to the proliferation of neuroblastoma cells and that inhibition of MCM2 and MCM10 expression may repress tumorigenesis. Therefore, these experiments confirm the dependence of MCM2 and MCM10 in neuroblastoma cells, and can be cellular targets for the development of drug therapy.

### 2.3. Inhibition of MCM2 and MCM10 for Neuroblastoma Therapy

**Figure 3:**
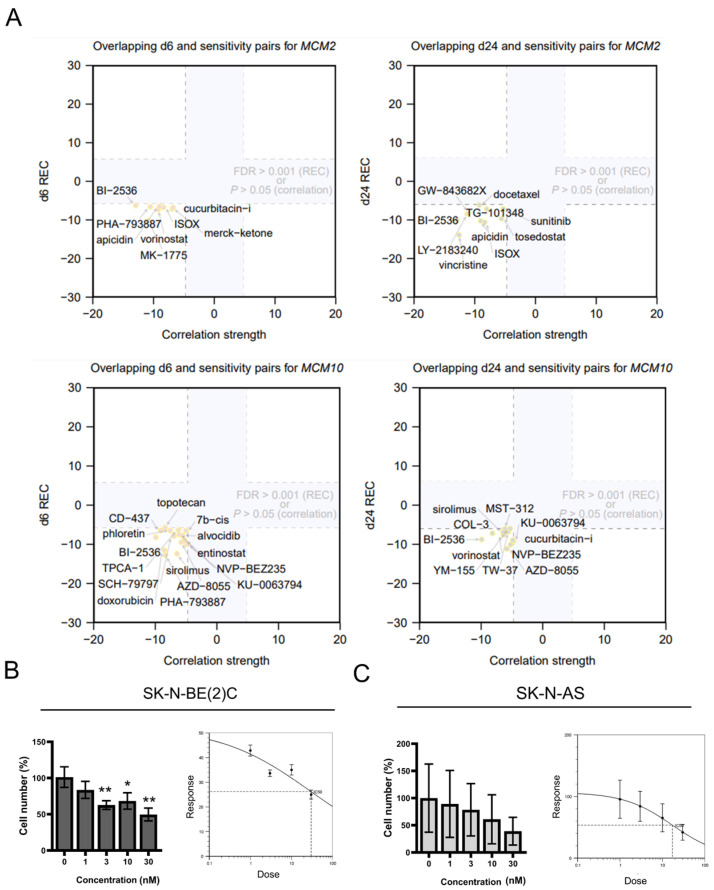
Discovery of compounds targeting MCM2 and MCM10: (**A**) Comparison between gene–sensitivity (*x*-axis) and perturbation–gene relationships (*y*-axis; REC analysis of perturbation profiles for 6 (d6) or 24 (d24) h) for genes MCM2 and MCM10 and small molecules. High expression was correlated with sensitivity to BI-2536 for both d6 and d24 comparison (n = 8 for d6 and n = 10 for d24; false discovery rate (FDR) ≤ 0.001 for REC analysis; *p* ≤ 0.05 for sensitivity correlation analysis). (**B**,**C**) Cell viability assay of SK-N-BE(2)C (**B**) and SK-N-AS (**C**) cells treated with BI-2536. Cell death was positively correlated with increasing concentrations of BI-2536 (from 0 to 30 nM). The half maximal inhibitory concentration (IC_50_) values were 28.23 and 17.30 nM for SK-N-BE(2)C and SK-N-AS cells, respectively (calculated and visualized using the IC_50_ Calculator (https://www.aatbio.com/tools/ic50-calculator; (accessed on 28 April 2020)). * *p* < 0.05, ** *p* < 0.01, from two-tailed unpaired Student’s *t*-test to determine whether the mean value of a given group is equal to the corresponding DMSO alone group.

We used an approach as previously described [16] to evaluate the recurrent expression of MCM2 and MCM10 genes under perturbations across 10 cancer cell lines using data from the LINCS L1000 data set. This led to a score denoted as REC (mRNA RECurrence across cell types), for which compound-induced upregulation or downregulation of a gene is assigned a positive or negative value, respectively. We sought to identify compounds that are under a clinical trial or approved for a different disease as potential candidates for neuroblastoma therapy. We screened 2214 compounds from their perturbation files for 6 and 24 h (Tables S1–S4; including only corrected *p*-values (as false discovery rates, or FDRs) ≤10^−3^ for small-molecule perturbagens). The REC score of BI-2536 was among the top 30 negative REC scores for both MCM2 and MCM10 for 6 and 24 h (denoted as d6 and d24, respectively). Moreover, we overlapped the results from this REC analysis with the relationships between basal cell-line expression of MCM2 or MCM10 to drug sensitivity data obtained from the Cancer Therapeutics Response Portal (CTRP) data set. Our analysis revealed that downregulation of MCM2 and MCM10 genes was associated with relatively strong gene reversal effects and strong sensitivity to BI-2536 (Figure 3A). BI-2536 is a polo-like kinase 1 (PLK-1) inhibitor used for the treatments of leukemia, carcinoma, pancreatic neoplasms, and non-small-cell lung cancer. We treated two neuroblastoma cell lines with BI-2536: MYCN-amplified SK-N-BE(2)C and MYCN-non-amplified SK-N-AS. The half maximal inhibitory concentration (IC_50_) at 24 h after treatment with BI-2536 was 28.23 and 17.30 nM, respectively, using a gradient concentration of 0, 1, 3, 10, 30, and 100 nM to interpolate the results (Figure 3B,C).

### 2.4. Cytotoxicity of BI-2536 in Neuroblastoma Cell Lines

**Figure 4:**
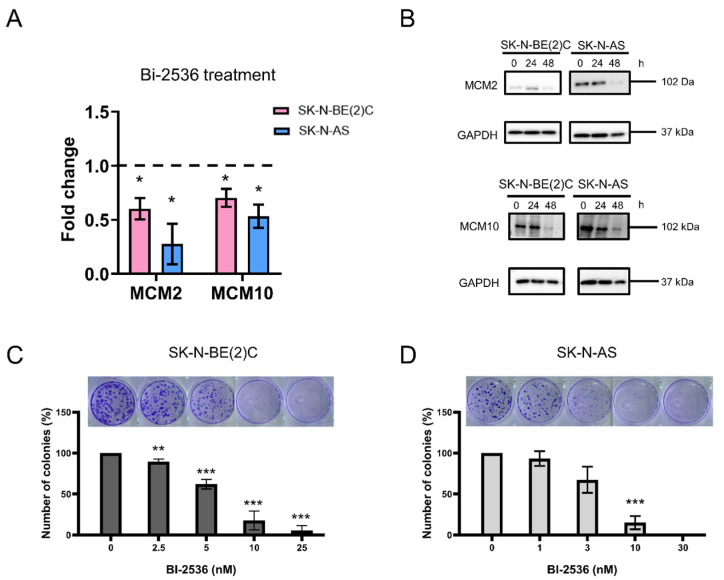
BI-2536 inhibited the expression of MCM2 and MCM10 and the proliferation of neuroblastoma cells: (**A**) MCM2 and MCM10 mRNA expression was decreased by BI-2536. The cells were treated with the half maximal inhibitory concentration (IC_50_) of BI-2536 for 24 h. * p < 0.05 from one-sample *t*-test to determine whether the group mean value differs from 1. (**B**) The protein levels of MCM2 and MCM10 were decreased in SK-N-BE (2) C and SK-N-AS cells following treatment with BI-2536 for 24 or 48 h; glyceraldehyde-3-phosphate dehydrogenase (GAPDH) was used as an internal control. The cells were treated with the IC_50_ of BI-2536 for 24 and 48 h. (**C**,**D**) BI-2536 reduced proliferation in neuroblastoma cells. The number of colonies was significantly reduced by >50% after treatment with 10–30 nM BI-2536 in SK-N-BE (2) C and SK-N-AS cells. ** *p* < 0.01, *** *p* < 0.001 from two-tailed unpaired Student’s *t*-test to determine whether the mean value of a given group is equal to the corresponding DMSO alone group.

Consistent with our predictions (Figure 3A), quantitative real-time polymerase chain reaction (qRT-PCR) assay showed that the mRNA expression of MCM2 and MCM10 was downregulated by BI-2536 treatment for 24 h (Figure 4A). Similarly, the protein levels of MCM2 and MCM10 decreased at 48 h after treatment with BI-2536 in SK-N-BE(2)C and SK-N-AS cells (Figure 4B). The colony formation assay revealed that cell proliferation was also inhibited by BI-2536 treatment in a dose-dependent manner. The number of colonies had significantly decreased in SK-N-BE(2)C and SK-N-AS cells treated with 10 nM BI-2536 for 14 days (Figure 4C,D).

Literature has suggested that some compounds (e.g., 10058-F4 and 10074-G5) can inhibit MAX-MYC heterodimerization and induce growth inhibition, apoptosis, and differentiation in MYCN-amplified neuroblastoma cells [17], and some compounds can prolong survival of MYCN transgenic mice [18]. We investigated whether neuroblastoma cells differentiated following treatment with BI-2536. For this purpose, we determined the amount of neurite outgrowth after treatment with BI-2536 for 24 and 48 h using a light microscope. At both 24 and 48 h after treatment, we observed a lower proportion of differentiated cells with fewer neurites (Figure S8B, Supplementary Materials). This finding indicated that BI-2536 treatment may directly induce cell death without affecting cell differentiation. We also used qRT-PCR to investigate the expression of some differentiation markers GAP43, CRT, NSE2, and NEFH after BI-2536 treatment. The expression of these marker genes is expected to be upregulated during cell differentiation; however, our results revealed that the changes in their expression levels were not significant (Figure S8C). Therefore, our results suggest that BI-2536 treatment did not induce neuroblastoma cell differentiation.

### 2.5. Gene Set Enrichment Analysis of BI-2536-Induced mRNA Recurrences

**Figure 5:**
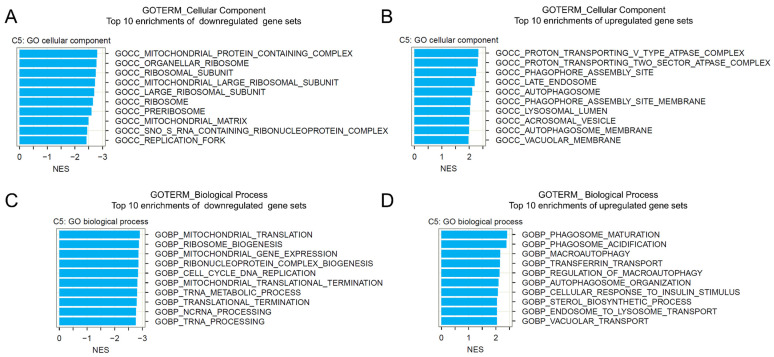
Gene set enrichment analysis (GSEA) of BI-2536-induced mRNA recurrences: Recurrence (REC) gene list, Gene Ontology (GO), Cellular Component (CC), and Biological Process (BP) gene sets were used for the GSEA analysis. The top 10 downregulated and upregulated CC (**A**,**B**) and BP (**C**,**D**) terms are shown (with false discovery rate (FDR) *q*-values of <0.25 from the GSEA analysis).

We further performed gene set enrichment analysis (GSEA) of BI-2536-induced mRNA recurrences (obtained from the REC analysis) to investigate the functions associated with BI-2536 treatment in cancer. The Gene Ontology (GO) Biological Process (BP) and Cellular Component (CC) gene set collections were used for the analysis. We found that the most significantly enriched GO CC terms involved mitochondria, ribosomes, and autophagy-associated organelles (Figure 5A,B). The most significant downregulated GO BP gene sets involved mitochondrial translation, mitochondrial gene expression, and cell cycle and DNA replication (Figure 5C), whereas the most significant upregulated gene sets involved phagosome maturation and macro-autophagy (Figure 5D). We also note downregulation of cell cycle DNA replication process in which MCM2 and MCM10 are involved after BI-2536 treatment, consistent with the major effects of the drug. Interestingly, several gene sets relevant to mitochondrial protein subunits were downregulated by BI-2536 treatment. Mitochondria, as the power house of a cell, can adapt to metabolic and other signals and maintain homeostasis by altering their morphology. Numerous mitochondrial functions have been linked to their morphology [19].

### 2.6. BI-2536-Induced Mitochondrial Fusion in Neuroblastoma Cells

**Figure 6:**
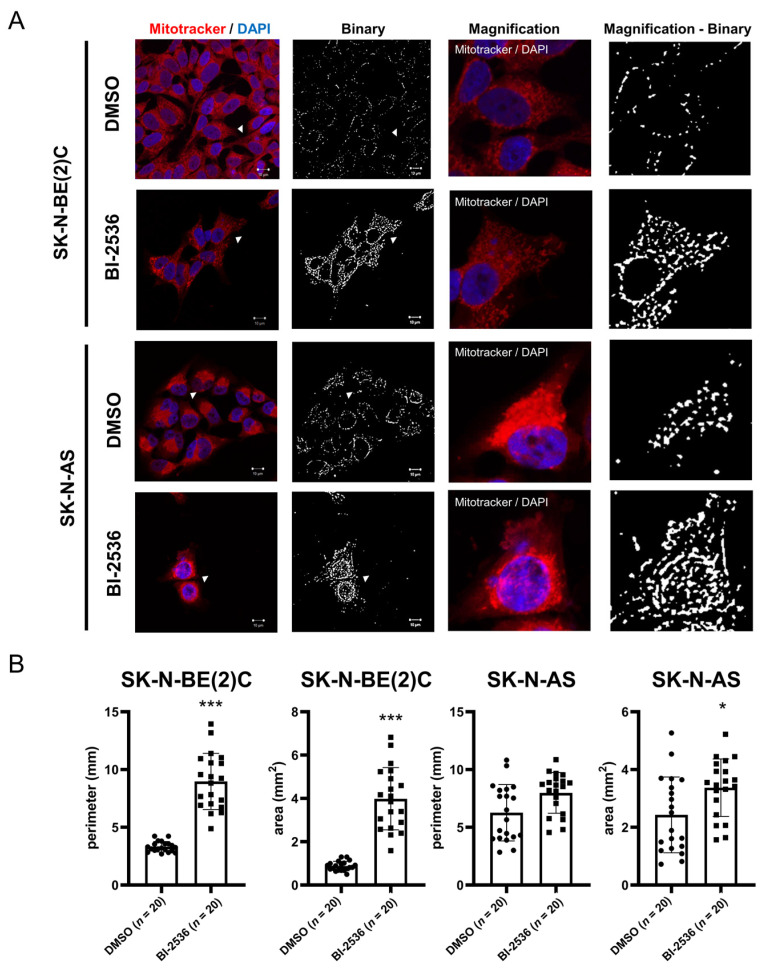
Immunocytochemistry showing mitochondrial dynamics after treatment of neuroblastoma cells with BI-2536 for 24 h: (**A**) The nucleus and mitochondria were stained using 4′,6-diamidino-2-phenylindole (DAPI) and MitoTracker^®^ (Thermo Fisher Scientific). Transformation to binary photography was performed using ICY ver. 2.1.0.0 software (http://icy.bioimageanalysis.org/; (accessed on 21 March 2021)). The mitochondrial perimeters and areas significantly increased after treatment, indicating that mitochondria in SK-N-BE (2) C and SK-N-AS cells fused and elongated after treatment with BI-2536 for 24 h. (**B**) Quantified data from the immunocytochemistry analysis. * *p* < 0.05, *** *p* < 0.001 from two-tailed unpaired Student’s *t*-test to determine whether the mean value of a given group is equal to the corresponding DMSO alone group.

Given the enrichments of mitochondrion-related terms (Figure 5)*,* we next determined whether mitochondrial functions are perturbed under BI-2536 treatment by focusing on mitochondrial dynamics as the overall condition of the mitochondria and cell cycle. We analyzed two parameters, perimeter and area, to define mitochondrial fusion and fission. At 24 h after BI-2536 treatment, the mitochondria of SK-N-BE(2)C and SK-N-AS cells fused and elongated, with an observed increase in perimeter and area (Figure 6). These results suggested that BI-2536-treated cells may experience stress; thus, cells stop the process of mitosis, and mitochondria fuse to produce more energy against BI-2536-induced cellular stress.

### 2.7. BI-2536-Induced Cell Cycle Arrest at the G2/M Phase and Apoptosis

**Figure 7:**
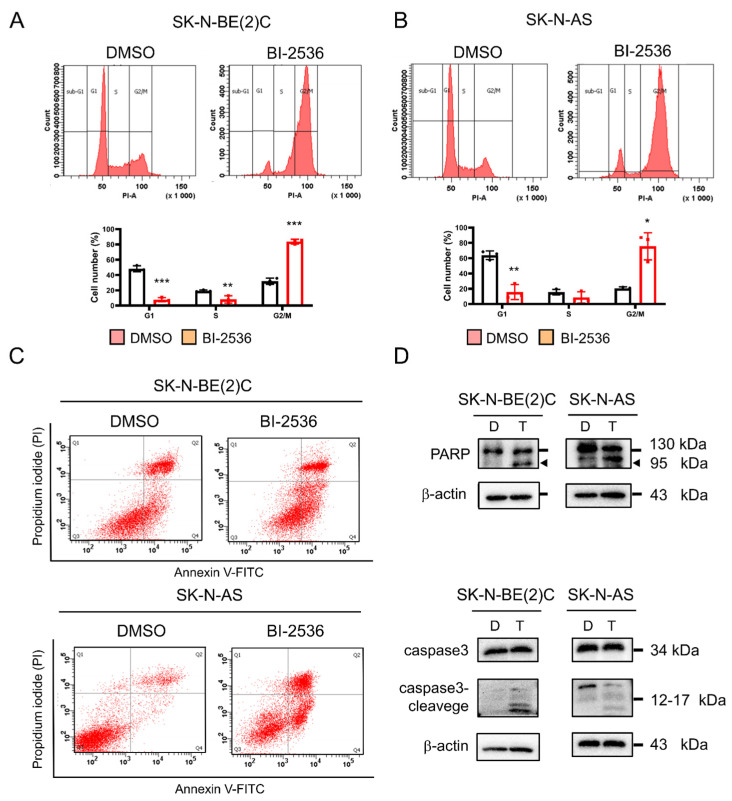
BI-2536-induced G2/M arrest and apoptosis in neuroblastoma cells: (**A**) SK-N-BE (2) C and (**B**) SK-N-AS cell cycle analysis was conducted using flow cytometry. At 24 h after treatment with BI-2536, >70% of the cells were in the G2/M phase. The cells were treated with the half maximal inhibitory concentration (IC_50_) of BI-2536 for 24 h. Quantitative data on the cell cycle phase for each cell line are shown in below. * *p* < 0.05, ** *p* < 0.01, *** *p* < 0.001 from two-tailed unpaired Student’s *t*-test to determine whether the mean value of a given group is equal to the corresponding DMSO alone group. (**C**) Cells in Q2 and Q4 represent late and early apoptosis, respectively; both increased after treatment with BI-2536. (**D**) Immunoblot analysis for apoptotic markers in BI-2536-treated neuroblastoma cells. The cleaved forms of poly-ADP ribose polymerase and caspase 3 (approximately 95 kDa and 12–17 kDa, respectively) were detected in SK-N-BE(2)C and SK-N-AS cells. The cells were treated with the IC_50_ of BI-2536 for 24 h. (D: DMSO, T: BI-2536).

SK-N-BE(2)C and SK-N-AS cells were examined for changes in cell cycle after treatment with BI-2536 for 24 h. The cell cycle was arrested in the G2/M phase (Figure 7A,B), consistent with on-target inhibition of PLK-1 by BI-2536. PLK-1 is involved in the establishment and separation of the spindle during mitosis. Moreover, knockdown of PLK-1 would result in a G2/M arrest phenotype. However, knockdown of MCM2 [20] or MCM10 [21] would lead to cell cycle arrest in the G1 phase. Therefore, BI-2536 may have a major function in the inhibition of PLK-1 and a minor function in DNA replication and other processes. 

Manipulation of the cell cycle can either induce or prevent apoptosis [22]. In addition, high levels of reactive oxygen species may lead to apoptosis [23], and these levels increase during mitochondrial fusion. Thus, we investigated cell apoptosis after treatment with BI-2536. BI-2536-induced apoptosis in both MYCN-amplified and MYCN-non-amplified cell lines (Figure 7C). Western blotting was used to detect the levels of the apoptosis markers cleaved caspase 3 and poly-ADP ribose polymerase (PARP) (Figure 7D).

## 3. Discussion and Conclusions

DNA replication is a tightly controlled biological process that is essential for cell division, wound tissue repair, and asexual reproduction [24,25]. Unregulated DNA replication leads to gene mutation and the development of cancer [26]. The process of DNA replication is initiated with the unwinding of dsDNA by MCM2-7. Subsequently, DNA polymerase binds to single-stranded DNA to initiate the complementary nucleic acid assembly and, finally, the extension process terminates at a stop codon [27,28]. MCM10 is also a member of the MCM family; however, it is involved in the recognition of the replication rigin site and recruitment of other factors, rather than functioning in the DNA helicase hexamer [29]. Nevertheless, the functional mechanism of MCM10 remains elusive. It has been reported that MCM2 is a proliferation marker in numerous types of cancer [30,31,32]. Moreover, high expression of MCM10 has been associated with poor outcomes in breast cancer and urothelial carcinoma [33,34]. 

In this study, we used a gene expression-based bioinformatic method for drug screening and subsequently identify BI-2536 for decreasing the DNA replication factors MCM2 and MCM10 and inducing neuroblastoma cell death. BI-2536 is an inhibitor of PLK-1. Therefore, we hypothesized that our experimental findings may be influenced by the PLK-1 knockdown phenotype. Indeed, in our experiments, the cell cycle was arrested in the G2/M phase. However, direct knockdown of MCM2 [20] or MCM10 [22] may lead to cell-cycle arrest in the G1 phase. Thus, our results indicate a PLK-1 inhibition phenotype. According to our data, we propose that BI-2536 plays a major role in the regulation of PLK-1 and a minor role in the regulation of MCM2 and MCM10.

Currently available drugs have been used in previous studies to target MCM2 and MCM10. Lovastatin, a lipid-lowering medicine used for the treatment of high levels of blood cholesterol and cardiovascular disease, induces cell cycle arrest and apoptosis, and inhibits cell growth in numerous types of cancer [35,36]. Zhang et al. used lovastatin to target MCM2 in non-small cell lung carcinoma. Lovastatin induces cell cycle arrest in the G1 phase and triggers apoptosis, reduces proliferation, and decreases the mRNA and protein levels of MCM2 [37]. Suramin, an anti-parasite medication, and its analogues can block the binding of MCM10 to dsDNA and reduce the replication products [38]. Hence, MCM2 and MCM10 can be target proteins for cancer therapy.

Mitochondrial fusion protects mitochondria through sharing of protein contents and mitochondrial DNA repair factors. In contrast, mitochondrial fission favors appropriate division into daughter cells during mitosis and separates damaged components, thus inducing mitophagy. The results of our GO analysis showed that the upregulated genes were related to autophagy. In general, mitophagy is dependent from autophagy [39]. Interestingly, mitochondria are degraded by autophagosome segregation and lysosomal fusion [40]. Therefore, mitochondria can be regarded as a substrate of autophagy. Nevertheless, according to our experiments, mitochondria fused following treatment with BI-2536. We reasoned that BI-2536 treatment induced mitochondrial fusion such that the treated cells transcriptionally activated mitochondrial fission genes as a compensatory mechanism in response to this stress (Tables S5 and S6, Supplementary Materials). 

## 4. Materials and Methods

### 4.1. Kaplan–Meier Curves of Overall Survival of Patients with Neuroblastoma

Two published neuroblastoma data sets (Versteeg-88-MAS5.0-u133p2 and SEQC-498-RPM-seqcnb1) were selected to analyze the overall survival of patients through the Kaplan–Meier method by the indicated gene expression levels (cutoff values selected using a scan model) across different stages of neuroblastoma. The Kaplan–Meier curves of overall survival were performed in the R2 platform (http://r2.amc.nl; (accessed on 28 May 2020)).

### 4.2. RNA Interference

SK-N-BE(2)C and SK-N-AS cells (5 × 10^5^) were seeded in 6-cm plates. After incubating for 24 h, each well of cells was transfected with 25 nM siRNA, either on-target against MCM2 or MCM10 or a non-targeting pool used as negative control (Dharmacon, Lafayette, CO, USA). The lipofectamine RNAiMAX transfection reagent was used in serine-free Dulbecco’s modified Eagle’s medium (DMEM, Thermo Scientific, Waltham, MA, USA). The efficiency of the transient silencing was validated based on the protein levels determined through Western blotting at 24, 48, and 72 h post transfection.

### 4.3. Drug Prediction

In drug prediction, we analyzed the differential expression of genes under perturbations in 10 cancer cell lines (i.e., A357, A549, HA1E, HCC515, HEPG2, HT29, MCF7, NPC, PC3, and VCAP) from the LINCS L1000 data set. To investigate the relationship between gene expression and perturbations, we used the REC score representing the degree of gene expression regulation by a given perturbation. This method has been previously described [16,41]. 

### 4.4. Cell Culture

Neuroblastoma cell lines SK-N-BE(2)C and SK-N-AS were obtained from the American Type Culture Collection (ATCC, Manassas, VA, USA) and maintained in DMEM (Thermo Scientific, Waltham, MA, USA) at pH 7.3–7.4 and supplemented with 10% fetal bovine serum (FBS, Thermo Scientific, MA, USA). In all experiments, cells were cultured at 37 °C with 5% CO_2_.

### 4.5. Drug Treatment

PLK-1 inhibitor BI-2536 (17385; Cayman Chemical Company, Ann Arbor, MI, USA) was dissolved in dimethyl sulfoxide (DMSO; Sigma-Aldrich, Burlington, MA, USA) to 50 mM as stock. BI-2536 was further diluted to 50 μM in DMEM for the treatment of cells. Each neuroblastoma cell line was treated with the IC_50_ dosage of BI-2536 for 24 or 48 h; an equal volume of DMSO was added to the control sample.

### 4.6. RNA Extraction and Reverse Transcription

Total mRNA was isolated from cells using the TRIzol reagent (Thermo Fisher, Waltham, MA, USA). After addition of chloroform, the lysate was mixed through gentle vortexing and centrifuged at 12,000× *g* for 20 min at 4 °C. In accordance with the Direct-zol™ RNA Miniprep instructions, an equal volume of ethanol (≥99.8%; Sigma-Aldrich, Burlington, MA, USA) was added to the supernatant and thoroughly mixed. The mixture was transferred to a Zymo-Spin™ IIC Column with a Collection Tube and centrifuged at 12,000× *g* for 30 s. DNase I was added directly to the column matrix and incubated for 15 min at room temperature to digest DNA. RNA Pre Wash buffer and RNA Wash buffer wash were used twice, and the lysate was centrifuged for 2 min to completely remove the buffer. DNase/RNase-free water was used to elute the RNA. RNA of quality between 1.8 and 2.0 260/280 optical density (O.D.) was reverse transcribed using a Revert H minus First Strand cDNA Synthesis Kit (Thermo Fisher, Waltham, MA, USA). RNA (500 ng) was used as template and reacted with oligo dT (1 μL) for 5 min at 60 °C. The reaction included 5X Reaction Buffer (4 μL), 10 mM deoxynucleoside triphosphate (2 μL), RiboLock RNase Inhibitor (1 μL), and RevertAid H Minus M-MuL V Reverse Transcriptase (200 U/μL) at 4 °C. Subsequently, reverse transcription was performed at 42 °C for 60 min. Thereafter, the buffer was heated at 70 °C for 5 min to deactivate the enzyme.

### 4.7. qRT-PCR

The IQ SYBR Green Supermix (Bio-Rad, Hercules, CA, USA; 10 μL), specific gene forward and reverse primers (10 μM; 0.4 μL), double-distilled water (ddH_2_O; 8.2 μL), and cDNA (1 μL) were placed into the qRT-PCR plate. The primer sequences are shown in Table S7 (Supplementary Materials). The procedure was initiated with denaturation at 95 °C for 5 min, followed by 40 cycles of denaturation at 95 °C for 10 s and annealing at 60 °C for 30 s. The reaction was performed using a CFX Connect system (Bio-Rad, Hercules, CA, USA). The mRNA expression was measured using Bio-Rad CFX Maestro (Bio-Rad, Hercules, CA, USA).

### 4.8. Cell Lysis and Protein Extraction

Cells were washed thrice with phosphate-buffered saline (PBS). Radioimmunoprecipitation assay buffer (50 Mm Tris-HCl, pH 7.4, 500 μL; 1% NP40, 100 μL; 0.1% sodium dodecyl sulfate, 100 μL; 150 mM NaCl 4.3 mL; 0.5% sodium deoxycholate, 5 mL) containing 10% Protein Inhibitor Cocktail (BioShop, Burlington, ON, Canada) and Phosphatase Inhibitor Cocktail I and II (BioShop, Burlington, ON, Canada) was used to lyse the cells, which were subsequently sonicated for 2–3 min on ice. The samples were centrifuged at 16,000× *g* for 20 min at 4 °C, and the supernatant was collected. The total protein levels were quantified using a BCA protein assay kit (Thermo Scientific, Waltham, MA, USA) for 15 min at 65 °C, and the O.D. 570 value was detected using a Model 680 Microplate Reader (Bio-Rad, Hercules, CA, USA).

### 4.9. Western Blot Analysis

Protein (20 μg) with 1× protein assay dye reagent (Bio-Rad, Hercules, CA, USA) was separated using sodium dodecyl sulfate-polyacrylamide gels (8–10% lower gel) and transferred to a polyvinylidene membrane (Merck Millipore, Burlington, MA, USA). The membrane was blocked with 5% fat-free milk/PBS with Tween-20 (PBST) (anti-rabbit MCM2 and anti-mouse beta-actin antibody), 2% bovine serum albumin/PBST (anti-rabbit PARP antibody), and 2% bovine serum albumin/tris-buffered saline with Tween 20 (TBST) (anti-rabbit MCM10 antibody) for 1 h at room temperature. Subsequently, it was incubated with blocking buffer containing primary antibody against MCM2 (1:1000; GeneTex, Irvine, CA, USA), MCM10 (1:1000; Abcam), or PARP (1:1000; GeneTex, Irvine, CA, USA) overnight at 4 °C. The membrane was washed thrice with PBST/TBST and incubated with secondary anti-mouse or anti-rabbit antibody (1:10,000; Sigma-Aldrich, Germany) for 2 h. The membrane was washed again thrice with PBST/TBST. Enhanced chemiluminescence substrate (Merck Millipore, Burlington, MA, USA) and Fluro ChemM (Glossop, Derbyshire, UK) were used to detect the protein content. Finally, protein quantification was conducted using ImageJ software.

### 4.10. Colony Formation Assay

Cells (1 × 10^3^/well) were seeded and treated with the IC_50_ dosage of BI-2536 for 24 h. The medium was replaced every three days. The duration of the experiment was 14 days. The cells were washed twice with PBS and fixed with 100% methanol overnight. Colonies were stained using 0.1% crystal violet (Sigma-Aldrich, Burlington, MA, USA) in 30% methanol for 5 min; excess crystal violet was removed with ddH_2_O (three washes). The number of colonies was calculated using Microsoft Paint 3D ver.6.2009.30067.0 (Microsoft Corporation, Redmond, WA, USA).

### 4.11. Cell Differentiation and Calculation of Neurites

qRT-PCR was used to measure the relative expression levels of genes related to differentiation in cells treated with the IC_50_ dosage of BI-2536 for 24 h. Cells (1 × 10^5^) were seeded in 6 cm plates and treated with the IC_50_ dosage of BI-2536 for 24 h. Five fields per plate were randomly selected at 24 and 48 h. The number of neurites per differential cell per field was calculated using a light microscope (type 090 135 002; Leica) under 200× magnification.

### 4.12. Gene Set Enrichment Analysis

We performed GSEA as previously described [15] using the Gene Ontology (GO) Biological Process (BP) and Cellular Component (CC) gene set collections (MSigDB v7.4, C5 collection) with default settings (1000 sample-level permutations; the minimum and maximum gene set sizes for consideration were set to 10 and 500, respectively). The mRNA REC scores associated with BI-2536 treatment were used as the gene list for GSEA [15]. Gene sets with a false discovery rate (FDR) *q*-value < 0.25 were considered statistically significant.

### 4.13. Immunocytochemistry and Mitochondrial Image Analysis

Cells (5 × 10^5^) were seeded on an 18 × 18 mm cover glass (Deckgläser) in a 12-well plate and treated with the IC_50_ dosage of BI-2536 for 24 h. Mitochondria were stained with 1:10,000 MitoTracker^®^ (Thermo Fisher, Waltham, MA, USA); cells were incubated with serine-free DMEM at 5% CO_2_ and 37 °C for 20 min, and washed twice with PBS. Cells were fixed with 3.7% paraformaldehyde (Sigma-Aldrich, Burlington, MA, USA) for 15 min, washed twice with PBS, stained with DAPI (20 μL), covered using a glass slide, and maintained at 4 °C overnight. The procedure after staining was conducted in darkness. The fluorescence signal was detected using a laser scanning confocal microscope Zeiss LSM 780 with an Airyscan detector with a Plan Apochromat 63 x/1.4 oil objective. The results were adjusted using Zen 2010 (Zeiss LSM 780; Jena, Germany).

To evaluate mitochondrial dynamics, we overlapped the Z-stack of images using Zen 2010, and the images were analyzed using the software ICY ver. 2.1.0.0 (http://icy.bioimageanalysis.org/; (accessed on 21 March 2021)). Firstly, we set the mitochondrial channel (Channel 1) and adjusted the intensity of fluorescence signals to enhance weak signals. The protocol was set for customized programming, and we extracted the selected channel from imaged mitochondria. Secondly, the images were blurred for the network mitochondria using a Gaussian filter tool, and further loaded the results to K-means thresholder for automatic adjustment. Next, we used a spot detector with a polygon enclosing tool to circle the cell and set the 3-pixel detection parameter to 100 specificity. The final results showed the perimeter, area, and shape of each detected mitochondrion.

### 4.14. Cell Cycle Analysis

Cells treated with BI-2536 for 24 h were washed twice using PBS and detached using trypsin-ethylenediaminetetraacetic acid. The cells were washed again with PBS and centrifuged at 1200× *g* for 5 min at room temperature, twice. Subsequently, they were resuspended in PBS (300 μL), treated with ethanol (700 μL) (≥99.8%; Sigma-Aldrich, Burlington, MA, USA), and fixed overnight at 4 °C. The cells were centrifuged at 1200× *g* for 5 min at room temperature and the supernatant was discarded. A mixture (500 μL) containing 100 μg/mL RNase A (Thermo Fisher, Waltham, MA, USA), 0.1% Triton X-100 (Sigma-Aldrich, Burlington, MA, USA), and PBS was added to the cells, which were incubated with 5% CO_2_ for 30 min at 37 °C. Cells were stained with 5 μg /mL propidium iodide (Santa Cruz, Dallas, TX, USA) to evaluate the DNA content. Detection and analysis of cell cycle were conducted using a BD FACSCanto II instrument (BD Biosciences, Franklin Lake, NJ, USA).

### 4.15. Apoptosis Assay

Apoptosis assays were conducted using an Apoptosis Detection kit (BD Biosciences, Franklin Lake, NJ, USA). Cells treated with BI-2536 for 24 h were washed twice with PBS and detached using trypsin-ethylenediaminetetraacetic acid. Subsequently, the cells were centrifuged twice at 1200× *g*, for 5 min at room temperature. Cells (1 × 10^6^) were resuspended in PBS (100 μL); subsequently, Annexin V (2 μL) and propidium iodide (2 μL) were added in darkness, and the mixture was allowed to stand for 15 min at room temperature. Binding buffer (400 μL) was added to stop the reaction. Flow cytometry analysis was conducted using the BD FACSCanto II instrument (BD Biosciences, Franklin Lake, NJ, USA).

### 4.16. Statistical Analysis

All cell-based experiments were conducted thrice, and data are shown as mean ± standard deviation unless specified otherwise. An appropriate statistical test was performed for each relevant panel of the figures to assess the statistical significance between groups (specifically indicated on each figure legend).

## 5. Conclusions

In conclusion, our analysis revealed that BI-2536 treatment induces mitochondrial fusion, cell cycle arrest in the G2/M phase, and apoptosis in neuroblastoma cells. BI-2536 may exert multiple effects on neuroblastoma cell death through inhibition of MCM2 and MCM10. Therefore, BI-2536 is a potent and multifunctional drug for the treatment of neuroblastoma.

## Data Availability

Data is contained within the article or Supplementary Materials.

## Supplementary Materials

The following are available online at https://www.mdpi.com/article/10.3390/ph15010037/s1: Figure S1: MCM member gene expression across INSS stages in the Vesteeg-88 database; Figure S2: MCM member gene expression across INSS stages in the SEQC-498 database; Figure S3: The over-all survival of neuroblastoma patients stratified by expression of each of MCM member genes in the Versteeg-88 database; Figure S4: The over-all survival of neuroblastoma patients stratified by expression of each of MCM member genes in the SEQC–498 database; Figure S5: The overall survival of patients MYCN-amplified or MYCN-nonamplified tumor and stratified by expression of MCM2 or MCM10 in different databases; Figure S6: MCM2 and MCM10 expression across 35 cancer types and corresponding normal tissue; Figure S7: Protein expressions of Knockdown efficiency of MCM2 and MCM10 in two cell lines; Figure S8: Cell morphology and expression changes of differentiation markers after BI-2536 treatment in neuroblastoma cells; Table S1: Recurrently regulated MCM2 by perturbations (d24 recurrent pairs); Table S2. Recurrently regulated MCM2 by perturbations (d6 recurrent pairs); Table S3: Recurrently regulated MCM10 by perturbations (d24 recurrent pairs); Table S4: Recurrently regulated MCM10 by perturbations (d6 recurrent pairs); Table S5: The recurrent relationships which significantly upregulated expression by BI-2536 treatment for 6 h; Table S6: The recurrent relationships which significantly upregulated expression by BI-2536 treatment for 24 h; Table S7. All sequences of primer used for qRT-PCR.
