# Supplementary material for: BI-2536 Promotes Neuroblastoma Cell Death via Minichromosome Maintenance Complex Components 2 and 10
Source: Pharmaceuticals (Basel). 2021 Dec 28;15(1):37. doi: 10.3390/ph15010037 (PMC8778242; doi:10.3390/ph15010037)
Supplement: Supplementary file 1 [file pharmaceuticals-15-00037-s001.zip › pharmaceuticals-1454153_Supplementary Material addition.pdf]

BI-2536 Promotes Neuroblastoma Cell Death via Minichromosome Maintenance Complex Components 2 and 10

Chiao-Hui Hsieh[1,†], Hsiang-Ning Yeh[1,†], Chen-Tsung Huang[2], Wei-Hsuan Wang[3], Wen-Ming Hsu[4], Hsuan-Cheng Huang[5,*], Hsueh-Fen Juan[1,2,3,6,*]

[1]Department of Life Science, National Taiwan University, Taipei 10617, Taiwan
[2]Graduate Institute of Biomedical Electronics and Bioinformatics, National Taiwan University, Taipei 10617, Taiwan
[3]Genome and Systems Biology Degree Program, National Taiwan University and Academia Sinica, Taipei 11529, Taiwan
[4]Department of Surgery, National Taiwan University Hospital and National Taiwan University College of Medicine, Taipei, 10017, Taiwan
[5]Institute of Biomedical Informatics, Center for Systems and Synthetic Biology, National Yang Ming Chiao Tung University, Taipei 11221, Taiwan
[6]Center for Computational and Systems Biology, National Taiwan University, Taipei 10617, Taiwan
[†]Authors contributed equally to this work and share first authorship.
[*]Correspondence: Hsuan-Cheng Huang (hsuancheng@nycu.edu.tw); Hsueh-Fen Juan (yukijuan@ntu.edu.tw)

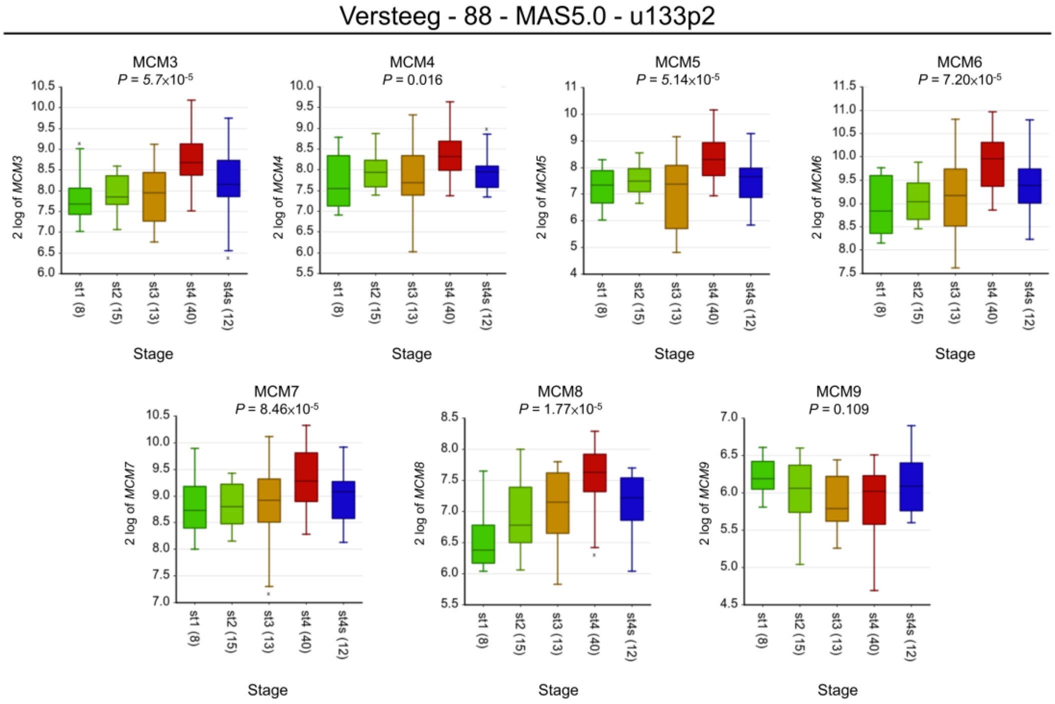

Figure S1. ***MCM*** **member gene expression across INSS stages in the Vesteeg-88 database**

Box-plots of *MCM3-9* expression in each INSS stage. The expression increases with the stage development, despite the stage 4S, is relatively low (generally better prognosis for this stage of disease). The x-axis represents the INSS stage with patient numbers shown in parentheses.

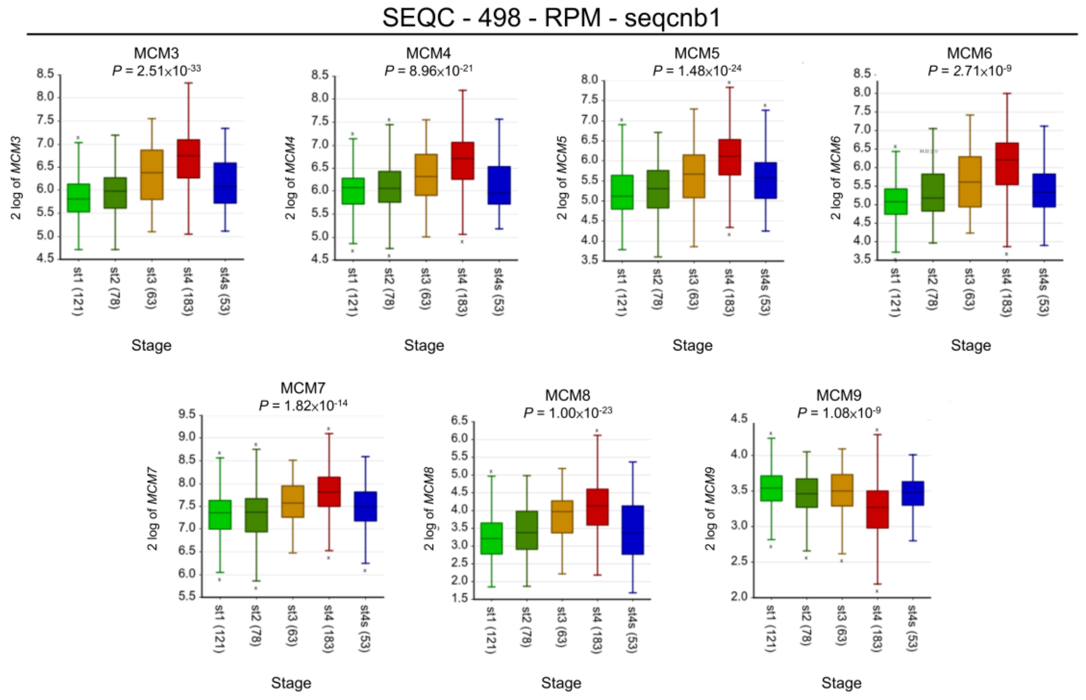

Figure S2. ***MCM*** **member gene expression across INSS stages in the SEQC-498 database**

Box-plots of *MCM3-9* expression in each INSS stage. The expression increases with the stage development, despite the stage 4S is relatively low (generally better prognosis for this stage of disease). The x-axis represents the INSS stage with patient numbers shown in parentheses.

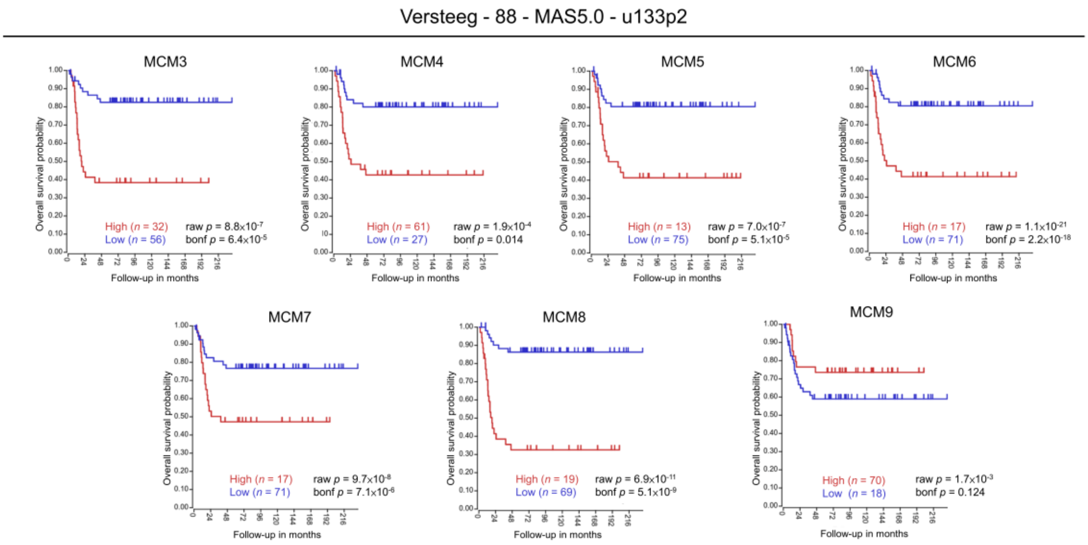

Figure S3. The over-all survival of neuroblastoma patients stratified by expression of each of *MCM* member genes in the Versteeg-88 database

The over-all survival was visualized by Kaplan Curve. High and low expression were denoted as red and blue, respectively. High expression of *MCM* genes correlated with poor survival except *MCM9*.

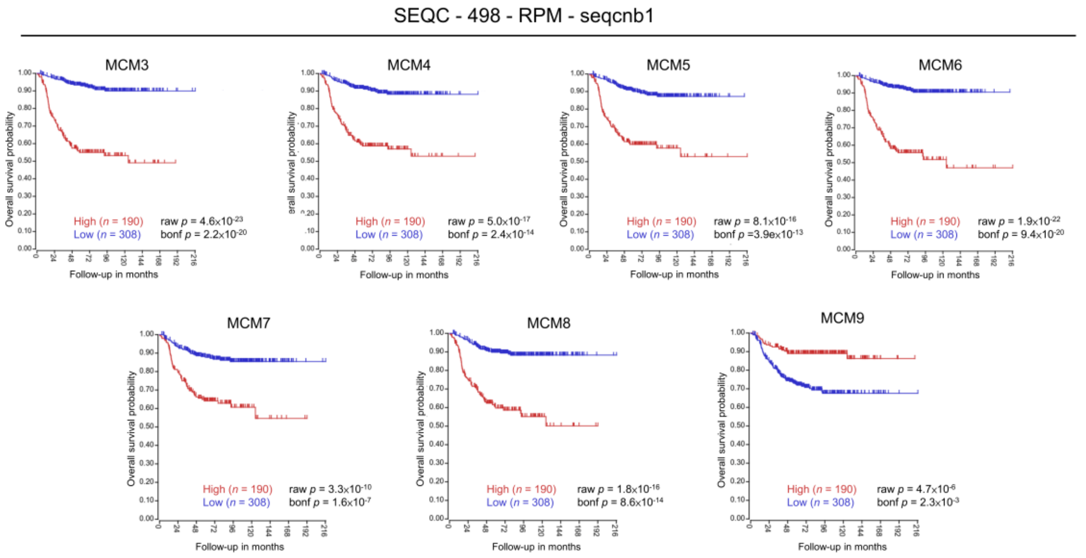

Figure S4. The over-all survival of neuroblastoma patients stratified by expression of each of *MCM* member genes in the SEQC–498 database

The over-all survival was visualized by Kaplan Curve. High and low expression were denoted as red and blue, respectively. High expression of *MCM* genes correlated with poor survival except *MCM9*.

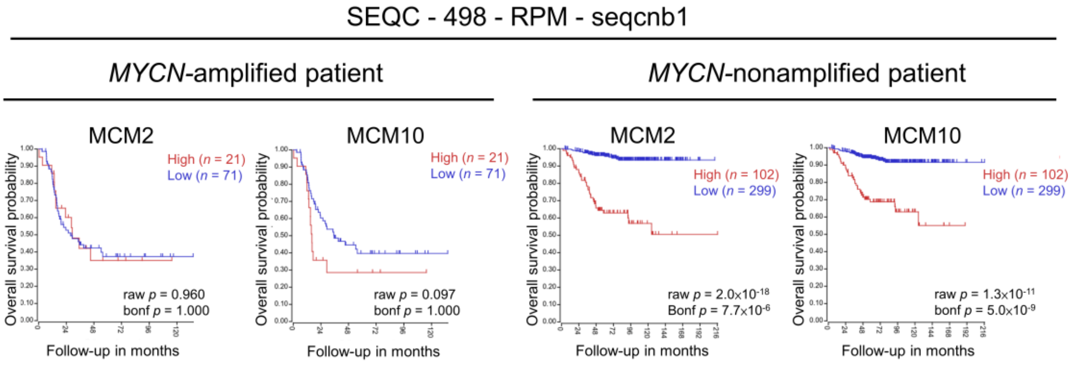

Figure S5. The overall survival of patients *MYCN*-amplified or *MYCN*-nonamplified tumor and stratified by expression of *MCM2* or *MCM10* in different databases

The over-all survival was visualized by Kaplan Curve. High and low expression were denoted as the color red and blue, respectively. In *MYCN*-amplified samples, the difference between survival rates was not significant. In *MYCN*-nonamplified tumors, high expression of *MCM2* and *MCM10* correlated with poor survival.

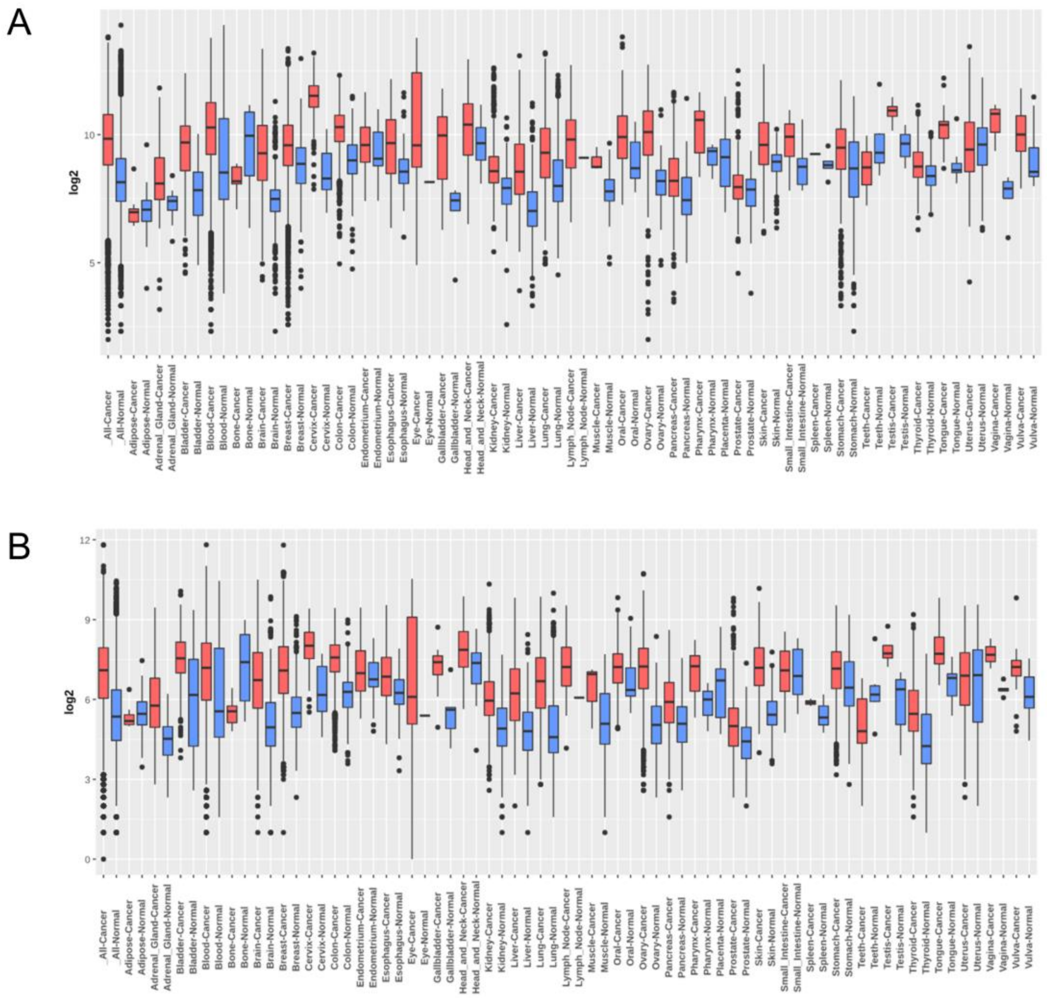

Figure S6. *MCM2* and *MCM10* expression across 35 cancer types and corresponding normal tissue

Expression of (A) *MCM2* and (B) *MCM10* across 35 tumor types. Using HG-U133_Plus_2 platform, the analysis was down by GENT2. The blue bars represent normal cells and red bars represent cancer cells.

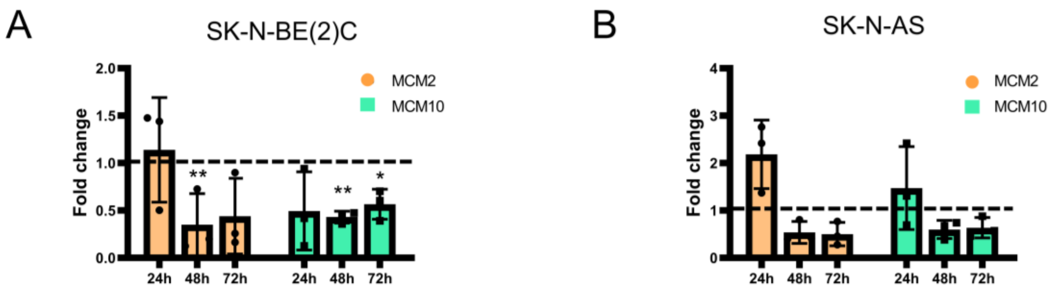

Figure S7. Protein expressions of Knockdown efficiency of MCM2 and MCM10 in two cell lines

The data represent the average protein expressions of MCM2 and MCM10 normalized by β-actin. **P* < 0.05, ***P* < 0.01 from two-tailed unpaired Student's *t*-test to determine whether the mean value of a given group is equal to the corresponding si*Ctrl* group alone group

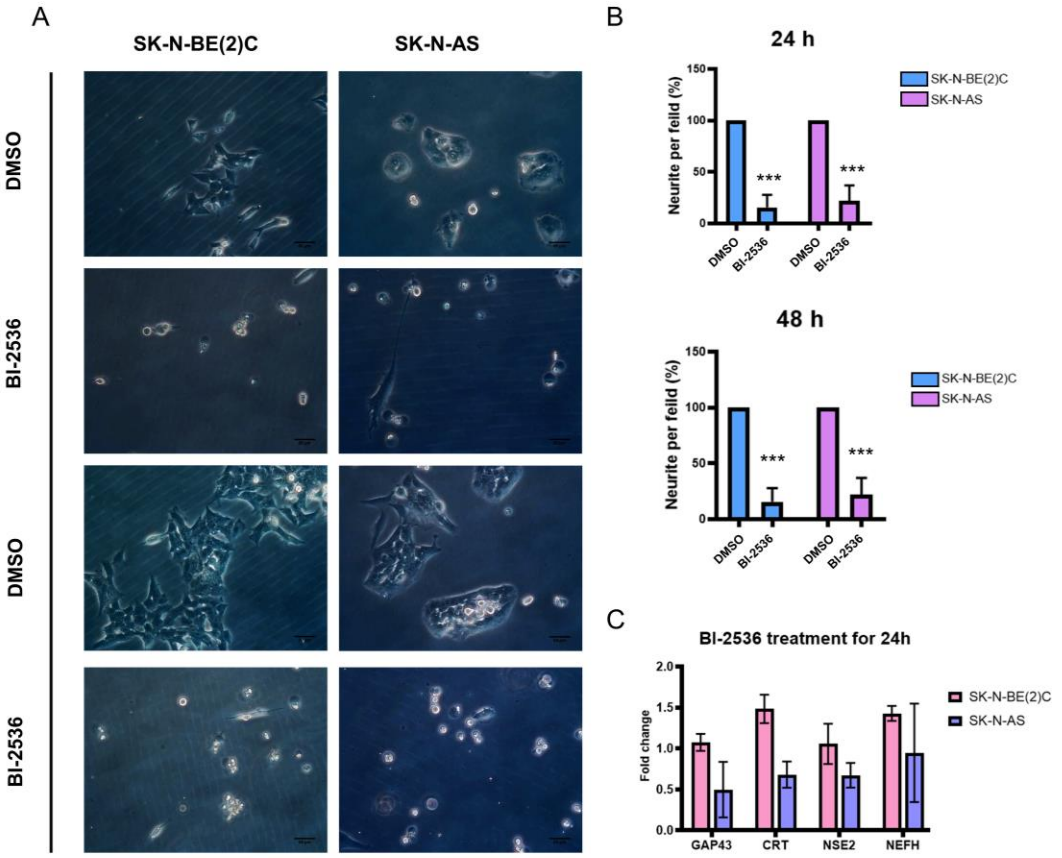

Figure S8. Cell morphology and expression changes of differentiation markers after BI-2536 treatment in neuroblastoma cells

(A) The number of neurites were decreased after BI-2536 treatment for 24 and 48 h. The cells floated from the bottom of culture dish and shrunk into ball shape, indicating cell death. (B) Statistical neurites number per cell per field reveal that neurites significantly decreased after BI-2536 treatment in SK-N-BE(2)C and SK-N-AS for 24 h. (C) Common differentiational marker genes: *GAP43*, *CRT*, *NSE2* and *NEFH* were checked by qRT-PCR. No significant change of differentiation markers was observed (upregulation of the marker genes indicate differentiation). The results show that cells did not undergo differentiation after BI-2536 treatment. *$P < 0.05$, **$P < 0.01$, ***$P < 0.001$ from two-tailed unpair student *t*-test..

Table S1. Recurrently regulated *MCM2* by perturbations (d24 recurrent pairs)

Perturbagen	REC	FDR	Relative rank									
			A375	A549	HA1E	HCC515	HEPG2	HT29	MCF7	NPC	PC3	VCAP
artesunate	–6.0573	8.56×10^{-4}	0.006	0.321	0.207	0.000	NA	0.021	0.001	NA	0.001	0.000
docetaxel	–6.1232	7.67×10^{-4}	NA	0.005	NA	NA	NA	NA	0.001	0.122	0.003	0.001
quiflapon	–6.1532	7.29×10^{-4}	0.014	0.185	0.123	0.001	NA	0.223	0.009	NA	0.003	0.014
CGK-733	–6.3290	5.45×10^{-4}	NA	0.014	0.227	0.011	0.198	NA	0.017	0.038	0.009	0.027
AG-592	–6.4041	4.80×10^{-4}	NA	0.020	0.135	0.004	NA	NA	0.005	0.064	0.003	0.137
KF-38789	–6.5298	3.88×10^{-4}	NA	0.044	0.068	0.074	NA	NA	0.064	0.011	0.001	0.099
IKK-16	–6.6782	3.02×10^{-4}	NA	0.025	NA	0.011	NA	NA	0.052	NA	0.012	0.005
ABT-751	–6.7639	2.61×10^{-4}	0.235	0.092	0.003	0.014	NA	0.010	0.116	NA	0.001	0.005
rottlerin	–6.8778	2.15×10^{-4}	0.061	0.206	0.074	0.002	NA	0.026	0.021	0.141	0.022	0.108
sunitinib	–7.1219	1.42×10^{-4}	NA	0.029	NA	NA	NA	NA	0.001	0.005	0.044	0.005
TG-101348	–7.1842	1.27×10^{-4}	0.013	0.199	NA	0.001	NA	0.007	0.021	NA	0.008	0.027
albendazole	–7.2407	1.16×10^{-4}	NA	0.057	0.095	0.015	NA	NA	0.004	0.053	0.001	0.058
selumetinib	–7.2982	1.05×10^{-4}	0.021	0.148	0.015	0.005	NA	0.003	0.021	NA	0.037	0.166
oxibendazole	–7.4911	7.57×10^{-4}	NA	0.151	0.055	0.003	NA	NA	0.021	0.007	0.009	0.008
vinorelbine	–7.5082	7.33×10^{-4}	NA	0.015	NA	NA	NA	NA	0.036	0.019	0.001	0.000
podophyllotoxin	–7.6482	5.74×10^{-5}	NA	0.058	0.005	0.245	0.031	NA	0.007	0.003	0.002	0.009
GW-843682X	–8.2286	2.12×10^{-5}	0.168	0.163	0.008	0.025	NA	0.005	0.002	0.083	0.000	0.002
IKK-2-inhibitor-V	–8.2617	2.00×10^{-5}	0.048	0.268	0.211	0.003	0.007	0.008	0.032	0.011	0.005	0.005
brefeldin-a	–8.2836	1.93×10^{-5}	0.014	0.011	0.120	0.174	NA	0.005	0.006	0.075	0.008	0.043
cyclosporin-a	–8.3180	1.82×10^{-5}	0.011	0.097	0.234	0.003	NA	0.043	0.016	0.007	0.020	0.011
LY-2183240	–8.4441	1.47×10^{5}	0.277	0.190	0.020	0.001	0.022	0.007	0.006	0.022	0.001	0.025

nocodazole	–8.7660	8.38×10^{-6}	NA	0.090	0.004	0.006	NA	NA	0.001	0.006	0.043	0.020
malonoben	–9.2423	3.63×10^{-6}	NA	0.021	0.061	0.003	0.010	NA	0.010	0.121	0.006	0.006
tosedostat	–9.6645	1.74×10^{-6}	0.021	0.009	0.130	0.001	NA	0.009	0.033	NA	0.004	0.002
PD-198306	–9.7274	1.56×10^{-6}	NA	0.013	0.060	0.015	NA	NA	0.011	0.016	0.009	0.001
methyl-2,5-dihydroxycinnamate	–9.9580	1.05×10^{-6}	NA	0.018	0.003	0.008	NA	NA	0.014	0.004	0.056	0.013
BI-2536	–10.1788	7.03×10^{-7}	0.010	0.004	0.051	0.002	NA	0.046	0.001	NA	0.004	0.067
apicidin	–10.2436	6.30×10^{-7}	0.057	0.070	0.039	0.002	0.136	0.006	0.004	0.040	0.042	0.004
ISOX	–10.6127	3.25×10^{-7}	0.037	0.059	0.023	0.002	NA	0.004	0.012	0.082	0.002	0.033
vinblastine	–12.5962	9.17×10^{-9}	NA	0.002	0.003	0.005	0.062	NA	0.003	0.008	0.002	0.006
vincristine	–13.9180	8.59×10^{-10}	NA	0.005	0.001	0.002	NA	NA	0.002	0.016	0.000	0.005

Table S2. Recurrently regulated *MCM2* by perturbations (d6 recurrent pairs)

Perturbagen	REC	FDR	Relative rank									
			A375	A549	HA1E	HCC515	HEPG2	HT29	MCF7	NPC	PC3	VCAP
palbociclib	–5.6381	$9.73{\times}10^{-4}$	0.010	0.001	0.512	0.001	0.198	0.002	0.002	NA	0.003	0.043
parthenolide	–5.6463	$9.60{\times}10^{-4}$	0.401	0.021	0.017	0.002	0.025	0.095	0.027	NA	0.183	0.090
radicicol	–5.6573	$9.41{\times}10^{-4}$	0.076	0.121	0.083	0.159	0.258	0.009	0.110	NA	0.099	0.002
prostratin	–5.8055	$7.29{\times}10^{-4}$	0.066	0.049	0.047	0.025	0.032	0.035	0.045	NA	0.021	0.466
scriptaid	–6.2381	$3.45{\times}10^{-4}$	0.286	0.007	0.076	0.174	0.022	0.012	0.019	0.030	0.176	0.196
BI–2536	–6.2539	$3.35{\times}10^{-4}$	0.085	0.019	0.011	0.126	0.358	0.015	0.043	NA	0.021	0.064
RO–28–1675	–6.3456	$2.86{\times}10^{-4}$	0.162	0.105	0.232	0.085	0.006	0.092	0.054	NA	0.013	0.010
WT–171	–6.3704	$2.74{\times}10^{-4}$	0.018	0.042	0.140	0.035	NA	0.111	0.011	NA	0.151	0.049
apicidin	–6.5125	$2.13{\times}10^{-4}$	0.228	0.159	0.205	0.027	0.123	0.017	0.028	0.112	0.029	0.021
PHA–793887	–6.5975	$1.84{\times}10^{-4}$	0.059	0.118	0.138	0.020	0.194	0.039	0.033	NA	0.100	0.018
niclosamide	–6.6376	$1.71{\times}10^{-4}$	0.158	0.062	0.038	0.004	0.149	0.043	0.042	0.300	0.105	0.009
trichostatin–a	–6.6620	$1.64{\times}10^{-4}$	0.184	0.019	0.090	0.169	0.113	0.046	0.048	0.216	0.026	0.008
ISOX	–6.7695	$1.36{\times}10^{-4}$	0.153	0.027	0.009	0.017	0.079	0.077	0.206	0.028	0.229	0.066
cucurbitacin–i	–6.8051	$1.27{\times}10^{-4}$	0.254	0.019	0.002	0.008	0.047	0.060	0.241	NA	0.013	0.002
dorsomorphin	–6.8147	$1.25{\times}10^{-4}$	0.219	0.017	0.074	0.040	0.004	0.082	0.076	NA	0.071	0.094
dacinostat	–7.0069	$8.90{\times}10^{-5}$	0.038	0.069	0.173	0.037	0.079	0.129	0.005	NA	0.100	0.014
NCH–51	–7.0480	$8.27{\times}10^{-5}$	0.188	0.098	0.023	0.043	NA	0.068	0.088	0.007	0.101	0.023
XMD–1499	–7.1139	$7.37{\times}10^{-5}$	0.015	0.005	0.110	0.004	0.046	0.002	0.006	NA	0.011	0.367

merck–ketone	–7.1667	6.70×10^{-5}	0.039	0.167	0.021	0.054	0.155	0.000	0.100	NA	0.018	0.062
vorinostat	–7.3214	5.09×10^{-5}	0.091	0.034	0.233	0.070	0.111	0.030	0.026	0.034	0.146	0.010
phorbol–12–myristat×1013–acetate	–7.5884	3.18×10^{-5}	0.108	0.067	0.026	0.011	0.008	0.026	0.080	NA	0.127	0.104
MK–1775	–10.2058	2.72×10^{-5}	0.146	0.010	0.017	0.022	0.039	0.023	0.006	NA	0.007	0.006

Table S3. Recurrently regulated *MCM10* by perturbations (d24 recurrent pairs)

Perturbagen	REC	FDR	Relative rank									
			A375	A549	HA1E	HCC515	HEPG2	HT29	MCF7	NPC	PC3	VCAP
suloctidil	–5.9732	9.82×10^{-4}	NA	0.024	0.121	0.015	NA	NA	0.005	0.184	0.037	0.039
YM–155	–5.9893	9.57×10^{-4}	0.024	0.150	0.081	0.032	NA	0.015	0.094	0.159	0.220	0.065
ZM–336372	–6.0067	9.30×10^{-4}	0.068	0.082	0.171	0.035	NA	0.004	0.010	NA	0.011	0.226
MST–312	–6.0531	8.62×10^{-4}	0.016	0.029	0.129	0.110	NA	0.013	0.023	NA	0.109	0.179
sorafenib	–6.0633	8.47×10^{-4}	0.185	0.020	NA	NA	NA	NA	0.036	0.003	0.006	0.001
selamectin	–6.1658	7.13×10^{-4}	NA	0.007	NA	0.003	NA	NA	0.002	NA	0.109	0.008
BIBU–1361	–6.1774	7.00×10^{-4}	0.001	0.221	0.062	NA	NA	0.005	0.000	NA	0.092	0.008
loperamide	–6.1989	6.75×10^{-4}	0.010	0.472	0.065	0.044	NA	0.001	0.002	0.066	0.013	0.010
lylamine	–6.2095	6.64×10^{-4}	NA	0.197	0.008	0.009	NA	NA	0.028	0.033	0.036	0.080
mycophenolat×10mofetil	–6.2247	6.48×10^{-4}	NA	0.038	0.120	0.044	NA	NA	0.004	0.022	0.046	0.122
BIBX–1382	–6.2715	5.99×10^{-4}	NA	0.005	0.282	0.002	NA	NA	0.000	0.021	0.023	0.025
triciribine	–6.2889	5.82×10^{-4}	NA	0.156	0.048	0.018	NA	NA	0.007	0.083	0.003	0.070
fluspirilene	–6.3306	5.44×10^{-4}	0.000	0.048	0.376	0.010	NA	0.027	0.002	0.041	0.118	0.092
LY–303511	–6.3380	5.37×10^{-4}	NA	0.037	0.011	0.018	NA	NA	0.088	0.002	0.170	0.051
sulconazole	–6.3527	5.23×10^{-4}	NA	0.132	0.147	0.007	NA	NA	0.003	0.002	0.050	0.034
T–98475	–6.3769	5.02×10^{-4}	NA	0.083	0.023	0.003	NA	NA	0.013	0.218	0.018	0.004
KU–0060648	–6.4173	4.69×10^{-4}	0.002	0.004	0.376	0.002	NA	0.078	0.005	NA	0.002	0.013
tyrphostin–A9	–6.4553	4.40×10^{-4}	NA	0.036	NA	NA	0.013	NA	0.006	0.134	0.009	0.022

geldanamycin	–6.4578	4.38×10^{-4}	0.023	0.030	0.361	0.003	0.017	0.226	0.002	0.042	0.029	0.179
linsitinib	–6.4645	4.33×10^{-4}	0.016	0.141	0.244	0.028	NA	0.008	0.001	NA	0.078	0.006
staurosporine	–6.5306	3.87×10^{-4}	0.025	0.007	0.267	0.017	0.005	0.077	0.009	NA	0.029	0.256
CCCP	–6.5470	3.77×10^{-4}	NA	0.008	0.094	0.001	NA	NA	0.001	0.190	0.033	0.019
amiodarone	–6.5978	3.46×10^{-4}	0.003	0.002	0.389	0.036	NA	0.002	0.030	0.023	0.092	0.078
neratinib	–6.6193	3.34×10^{-4}	0.008	0.095	NA	0.003	NA	0.027	0.009	NA	0.100	0.105
NNC–05–2090	–6.6815	3.01×10^{-4}	NA	0.180	0.084	0.003	NA	NA	0.001	0.046	0.011	0.009
triptolide	–6.7051	2.89×10^{-4}	0.009	0.030	0.025	0.117	NA	0.017	0.077	NA	0.010	0.209
lasalocid	–6.7274	2.78×10^{-4}	0.002	0.001	0.330	0.002	NA	0.036	0.083	0.178	0.006	0.008
atorvastatin	–6.7490	2.68×10^{-4}	0.001	0.087	0.075	0.003	0.115	0.205	0.000	0.355	0.005	0.008
malonoben	–6.7995	2.46×10^{-4}	NA	0.228	0.025	0.002	0.003	NA	0.002	0.198	0.001	0.004
TW–37	–6.8003	2.46×10^{-4}	0.003	0.007	0.158	0.024	NA	0.067	0.004	NA	0.041	0.177
purvalanol–a	–6.8423	2.29×10^{-4}	0.057	0.017	0.066	NA	NA	0.003	0.002	NA	0.006	0.167
sirolimus	–6.8500	2.26×10^{-4}	0.021	0.002	0.026	0.001	0.089	0.537	0.003	0.002	0.029	0.002
RS–17053	–6.8627	2.21×10^{-4}	NA	0.040	0.071	0.011	NA	NA	0.029	NA	0.000	0.041
bithionol	–6.8970	2.08×10^{-4}	NA	0.185	0.042	0.005	NA	NA	0.004	0.003	0.063	0.006
fostamatinib	–6.9012	2.07×10^{-4}	0.003	0.088	0.302	0.005	NA	0.001	0.029	NA	0.005	0.005
rhodomyrtoxin–b	–6.9904	1.78×10^{-4}	0.005	0.003	0.058	NA	NA	0.005	0.085	NA	0.001	0.145
VER–155008	–7.0659	1.56×10^{-4}	0.000	0.075	0.151	0.002	NA	0.143	0.003	NA	0.033	0.040
maprotiline	–7.0908	1.50×10^{-4}	0.038	0.029	0.053	0.003	NA	0.189	0.013	0.066	0.058	0.175
KO–143	–7.0978	1.48×10^{-4}	0.014	0.049	0.302	0.003	NA	0.006	0.002	NA	0.001	0.034
dorsomorphin	–7.1013	1.47×10^{-4}	0.002	0.183	0.151	0.009	NA	0.016	0.014	0.005	0.004	0.221

NVP–TAE684	–7.1072	1.46×10^{-4}	NA	0.008	NA	NA	NA	NA	0.006	0.026	0.019	0.027
CGP–71683	–7.1601	1.33×10^{-4}	NA	0.003	0.025	0.006	NA	NA	0.004	0.216	0.007	0.012
DG–041	–7.1660	1.32×10^{-4}	0.002	0.050	0.320	0.002	NA	0.012	0.002	NA	0.004	0.002
COL–3	–7.1931	1.26×10^{-4}	0.032	0.010	0.253	0.005	0.037	0.029	0.043	NA	0.008	NA
calcitriol	–7.2429	1.16×10^{-4}	0.172	0.050	0.025	0.001	NA	0.094	0.010	0.181	0.005	0.061
tunicamycin	–7.2924	1.06×10^{-4}	NA	0.007	NA	NA	NA	NA	0.012	0.036	0.017	0.007
perhexiline	–7.3452	9.73×10^{-5}	0.005	0.185	0.128	0.021	NA	0.011	0.004	NA	0.000	0.053
WY×10354	–7.4236	8.51×10^{-5}	NA	0.017	NA	NA	NA	NA	0.025	0.029	0.002	0.001
mitoxantrone	–7.5200	7.19×10^{-5}	0.004	0.003	0.015	NA	0.005	0.006	0.000	NA	0.004	0.315
FGIN–1–27	–7.5801	6.47×10^{-5}	0.026	0.065	0.108	0.060	NA	0.004	0.010	NA	0.003	0.113
vorinostat	–7.5940	6.31×10^{-5}	0.051	0.094	0.034	0.004	0.022	0.359	0.011	0.033	0.038	0.049
chlorpromazine	–7.6054	6.19×10^{-5}	0.142	0.001	0.043	0.004	NA	NA	0.000	0.086	0.098	0.009
nonoxynol–9	–7.6904	5.34×10^{-5}	NA	0.104	0.015	0.002	NA	NA	0.007	0.001	0.027	0.085
L–690488	–7.7691	4.66×10^{-5}	0.003	0.153	0.126	0.003	NA	0.007	0.028	NA	0.005	0.037
tyrphostin–AG–1478	–7.7823	4.56×10^{-5}	0.002	0.142	0.144	0.006	NA	0.013	0.000	0.177	0.001	0.029
NVP–AUY922	–7.8486	4.07×10^{-5}	0.001	0.020	0.252	0.014	NA	0.003	0.005	NA	0.030	0.011
WY×10125132	–7.8701	3.92×10^{-5}	NA	0.012	NA	NA	NA	NA	0.001	0.003	0.016	0.028
AKT–inhibitor–1–2	–8.0936	2.67×10^{-5}	0.005	0.083	NA	0.001	NA	0.095	0.022	NA	0.001	0.005
tosedostat	–8.1202	2.56×10^{-5}	0.021	0.164	0.118	0.004	NA	0.008	0.001	NA	0.006	0.001
BX–795	–8.3943	1.60×10^{-5}	0.001	0.144	0.082	0.003	NA	0.064	0.002	NA	0.006	0.001
PIK–90	–8.6119	1.10×10^{-5}	0.005	0.193	0.065	0.002	NA	0.003	0.002	NA	0.004	0.002
BI–2536	–8.7633	8.42×10^{-6}	0.003	0.106	0.027	0.023	NA	0.077	0.018	NA	0.007	0.017

torin–1	–8.7771	8.21×10^{-6}	NA	0.005	NA	NA	NA	NA	0.007	0.003	0.003	0.021
BMS–754807	–8.8381	7.36×10^{-6}	0.007	0.166	0.082	0.002	NA	0.001	0.001	NA	0.001	0.000
KU–0063794	–8.9604	5.95×10^{-6}	0.001	0.159	0.076	0.005	NA	0.008	0.003	NA	0.001	0.001
CGK–733	–9.1837	4.03×10^{-6}	NA	0.008	0.032	0.005	0.033	NA	0.001	0.086	0.009	0.071
cucurbitacin–i	–9.1949	3.95×10^{-6}	0.023	0.017	0.015	0.013	0.014	0.017	0.017	0.310	0.014	0.033
AKT–inhibitor–IV	–9.3745	2.88×10^{-6}	NA	0.029	NA	0.002	NA	0.006	0.006	NA	0.008	0.022
GR–127935	–9.4147	2.68×10^{-6}	NA	0.016	0.008	0.008	NA	NA	0.001	0.080	0.019	0.004
cytochalasin–b	–9.4461	2.54×10^{-6}	0.002	0.037	0.013	0.003	NA	0.054	0.017	NA	0.032	0.070
NVP–BEZ235	–10.0773	8.46×10^{-7}	0.002	0.013	0.231	0.001	NA	0.001	0.018	0.001	0.001	0.001
PI–828	–10.1610	7.25×10^{-7}	NA	0.009	0.018	0.002	NA	NA	0.003	0.067	0.006	0.002
artesunate	–10.2138	6.64×10^{-7}	0.003	0.053	0.075	0.004	NA	0.002	0.041	NA	0.001	0.002
IKK–16	–10.8060	2.27×10^{-7}	NA	0.001	NA	0.002	NA	NA	0.004	NA	0.008	0.001
AZD–8055	–11.1692	1.17×10^{-7}	0.003	0.064	0.054	0.008	NA	0.002	0.000	NA	0.004	0.001
ivermectin	–11.2949	9.33×10^{-8}	NA	0.014	0.016	0.001	NA	NA	0.000	NA	0.001	0.002
PD–198306	–11.3662	8.20×10^{-8}	NA	0.005	0.036	0.005	NA	NA	0.004	0.005	0.001	0.017
IKK–2–inhibitor–V	–11.6204	5.11×10^{-8}	0.003	0.012	0.005	0.003	0.005	0.002	0.002	0.203	0.002	0.038
niguldipine	–11.7793	3.85×10^{-8}	0.002	0.090	0.011	0.002	NA	0.005	0.001	NA	0.001	0.001
cyclosporin–a	–12.2548	1.68×10^{-8}	0.008	0.006	0.034	0.010	NA	0.003	0.005	0.095	0.003	0.001
rottlerin	–12.4102	1.28×10^{-8}	0.006	0.030	0.014	0.003	NA	0.003	0.004	0.066	0.002	0.032
niclosamide	–12.5238	1.05×10^{-8}	0.001	0.024	0.015	0.020	NA	0.013	0.004	0.058	0.001	0.020
TGX–115	–13.3308	2.50×10^{-9}	0.001	0.017	0.025	0.003	NA	0.003	0.020	NA	0.002	0.002
TG–101348	–14.1153	6.13×10^{-10}	0.002	0.008	NA	0.002	NA	0.004	0.002	NA	0.002	0.008

Table S4. Recurrently regulated *MCM10* by perturbations (d6 recurrent pairs)

Perturbagen	REC	FDR	Relative rank									
			A375	A5⁴9	HA1E	HCC515	HEPG2	HT29	MCF7	NPC	PC3	VCAP
ER–27319	–5.6230	9.99×10^{-4}	0.272	0.055	0.098	0.101	0.141	0.005	0.013	NA	0.034	0.196
wortmannin	–5.6481	9.57×10^{-4}	0.013	0.009	0.010	0.057	0.152	0.182	0.061	0.533	0.008	0.004
BAS–00535043	–5.6487	9.56×10^{-4}	0.037	0.136	0.130	0.014	NA	0.163	0.008	NA	0.050	0.149
importazole	–5.6590	9.38×10^{-4}	0.041	0.332	0.130	0.143	0.032	0.033	0.077	NA	0.021	0.086
oligomycin–a	–5.6651	9.29×10^{-4}	0.040	0.171	0.063	0.269	0.027	0.002	0.257	NA	0.060	0.001
KU–55933	–5.6755	9.12×10^{-4}	0.041	0.313	0.032	0.209	0.011	0.013	0.094	NA	0.016	0.156
ZSTK–474	–5.6888	8.92×10^{-4}	0.473	0.037	0.048	0.024	0.003	0.038	0.008	NA	0.168	0.002
FCCP	–5.7209	8.43×10^{-4}	0.083	0.208	0.015	0.158	0.030	0.013	0.007	NA	0.344	0.001
topotecan	–5.7377	8.19×10^{-4}	0.005	0.077	0.021	0.325	NA	0.117	0.013	NA	0.010	0.067
AG–14361	–5.7437	8.11×10^{-4}	0.015	0.174	0.057	0.167	0.043	0.038	0.005	NA	0.039	0.328
JAK3–Inhibitor–II	–5.7961	7.41×10^{-4}	0.018	0.072	0.132	0.129	0.040	0.121	0.125	NA	0.238	0.012
JNK–9L	–5.8458	6.80×10^{-4}	0.449	0.090	0.099	0.073	0.016	0.007	0.002	NA	0.022	0.026
WY×10354	–5.8826	6.38×10^{-4}	0.029	0.010	0.364	0.073	0.235	0.048	0.005	NA	0.005	0.040
temsirolimus	–5.9287	5.89×10^{-4}	0.046	0.012	0.124	0.004	0.424	0.011	0.003	NA	0.081	0.075
SRT–1720	–6.0061	5.15×10^{-4}	0.104	0.011	0.019	0.128	0.025	0.124	0.027	NA	0.224	0.171
PI–103	–6.0485	4.79×10^{-4}	0.002	0.034	0.071	0.009	0.439	0.078	0.033	0.295	0.002	0.003
BAPTA–AM	–6.0675	4.63×10^{-4}	0.060	0.043	0.049	0.084	0.024	0.013	0.018	NA	0.183	0.320
dactinomycin	–6.1258	4.19×10^{-4}	0.032	0.026	0.057	0.179	0.014	0.036	0.037	NA	0.027	0.358

ouabain	–6.1680	3.89×10^{-4}	0.019	0.018	0.166	0.171	0.013	0.029	0.181	NA	0.139	0.062
pyrvinium–pamoate	–6.2666	3.28×10^{-4}	0.006	0.045	0.399	0.054	0.090	0.053	0.001	NA	0.025	0.046
quiflapon	–6.3193	2.99×10^{-4}	0.138	0.091	0.013	0.058	0.007	0.071	0.038	NA	0.278	0.062
TPCA–1	–6.3316	2.93×10^{-4}	0.111	0.017	0.278	0.022	0.039	0.004	0.144	NA	0.110	0.024
triptolide	–6.3683	2.75×10^{-4}	0.022	0.074	0.007	0.096	0.065	0.003	0.065	NA	0.055	0.340
proscillaridin	–6.4163	2.52×10^{-4}	0.077	0.043	0.061	0.046	0.072	0.124	0.225	NA	0.090	0.016
XMD–16144	–6.4485	2.38×10^{-4}	0.028	0.019	0.134	0.024	0.136	0.118	0.146	NA	0.098	0.049
CD–437	–6.4545	2.36×10^{-4}	0.001	0.011	0.199	0.196	0.101	0.102	0.013	NA	0.092	0.023
phloretin	–6.4999	2.18×10^{-4}	0.000	0.388	0.042	0.063	0.007	0.021	0.129	NA	0.019	0.001
DG–041	–6.5020	2.17×10^{-4}	0.083	0.027	0.045	0.070	0.224	0.103	0.020	NA	0.138	0.023
7b–cis	–6.5260	2.08×10^{-4}	0.058	0.004	0.216	0.013	0.097	0.060	0.009	NA	0.226	0.032
JAK3–inhibitor–VI	–6.5602	1.96×10^{-4}	0.210	0.022	0.016	0.009	0.002	0.008	0.003	NA	0.111	0.303
PP–110	–6.5681	1.93×10^{-4}	0.012	0.340	0.039	0.004	0.193	0.004	0.049	NA	0.002	0.030
camptothecin	–6.6220	1.76×10^{-4}	0.005	0.036	0.056	0.271	0.130	0.069	0.001	NA	0.118	0.007
JNJ–7706621	–6.8654	1.14×10^{-4}	0.037	0.060	0.091	0.051	0.109	0.035	0.017	NA	0.085	NA
purvalanol–a	–6.8751	1.12×10^{-4}	0.002	0.006	0.049	0.006	NA	0.306	0.004	NA	0.064	0.003
perhexiline	–6.9292	1.02×10^{-4}	0.016	0.015	0.114	0.015	0.073	0.165	0.173	NA	0.059	0.025
AKT–inhibitor–IV	–7.0879	7.71×10^{-5}	0.002	0.102	0.127	0.061	0.146	0.068	0.014	NA	0.009	0.105
NVP–TAE684	–7.1031	7.50×10^{-5}	0.003	0.020	0.327	0.030	0.040	0.012	0.012	NA	0.110	0.035
PNU–74654	–7.1123	7.39×10^{-5}	0.009	0.079	0.029	0.291	0.073	0.061	0.055	0.111	0.055	0.053
AT–7519	–7.4105	4.35×10^{-5}	0.027	0.131	0.053	0.004	0.146	0.040	0.001	NA	0.112	0.066
KU–0060648	–7.4381	4.14×10^{-5}	0.001	0.185	0.050	0.075	0.110	0.069	0.024	NA	0.011	0.049

IKK–16	–7.5068	3.68×10^{-5}	0.100	0.006	0.016	0.095	0.271	0.014	0.001	NA	0.007	0.032
entinostat	–7.5444	3.43×10^{-5}	0.009	0.005	0.054	0.003	0.044	0.144	0.247	NA	0.004	0.030
terfenadine	–7.5531	3.38×10^{-5}	0.042	0.113	0.005	0.029	0.008	0.053	0.263	NA	0.005	0.020
mocetinostat	–7.5922	3.15×10^{-5}	0.105	0.015	0.110	0.044	0.005	0.033	0.191	NA	0.015	0.029
bisindolylmaleimide	–7.6039	3.09×10^{-5}	0.011	0.042	0.133	0.059	0.036	0.330	0.000	0.030	0.027	0.036
GDC–0941	–7.6077	3.07×10^{-5}	0.061	0.003	0.175	0.011	0.180	0.073	0.015	NA	0.004	0.019
TG–101348	–7.6397	2.89×10^{-5}	0.021	0.004	0.201	0.004	0.086	0.109	0.002	NA	0.005	0.104
alvocidib	–7.7541	2.36×10^{-5}	0.157	0.012	0.081	0.109	0.088	0.009	0.003	NA	0.044	0.025
SCH–79797	–7.9198	1.75×10^{-5}	0.055	0.040	NA	0.086	0.113	0.014	0.015	NA	0.001	0.030
CCCP	–7.9234	1.74×10^{-5}	0.007	0.046	0.039	0.104	0.002	0.032	0.129	NA	0.108	0.042
BI–2536	–8.2075	1.04×10^{-5}	0.001	0.004	0.043	0.002	0.079	0.008	0.116	NA	0.088	0.128
staurosporine	–8.2235	1.01×10^{-5}	0.023	0.007	0.047	0.013	0.010	0.018	0.098	0.124	0.073	0.217
NU–7441	–8.2533	9.60×10^{-6}	0.026	0.016	0.052	0.022	0.146	0.048	0.082	NA	0.026	0.049
CP466722	–8.3108	8.66×10^{-6}	0.029	0.006	0.078	0.197	0.040	0.007	0.004	NA	0.080	0.008
ZG–10	–8.4448	6.78×10^{-6}	0.036	0.009	0.021	0.056	0.031	0.024	0.110	NA	0.147	0.009
torin–1	–8.5790	5.31×10^{-6}	0.129	0.013	0.077	0.003	0.180	0.001	0.002	NA	0.002	0.009
CGP–60474	–8.6518	4.65×10^{-6}	0.037	0.132	0.144	0.023	0.005	0.014	0.009	NA	0.025	0.028
GSK–1059615	–8.8764	3.09×10^{-6}	0.001	0.002	0.091	0.091	NA	0.003	0.073	NA	0.002	0.002
CHEMBL–399379	–8.9985	2.47×10^{-6}	0.008	0.078	NA	0.039	0.044	0.011	0.024	NA	0.005	0.051
NVP–BEZ235	–9.0566	2.22×10^{-6}	0.001	0.013	0.013	0.002	0.112	0.003	0.014	0.240	0.008	0.098
SB–218078	–9.0579	2.22×10^{-6}	0.005	0.012	0.008	0.075	0.004	0.005	0.019	NA	0.031	0.207
palbociclib	–9.1105	2.01×10^{-6}	0.001	0.011	0.014	0.010	0.003	0.004	0.003	NA	0.003	0.287

tyrphostin–AG–1478	–9.2156	1.67×10^{-6}	0.228	0.001	0.018	0.002	0.036	0.006	0.042	NA	0.009	0.002
pidorubicine	–9.2582	1.54×10^{-6}	0.043	0.019	0.013	0.089	0.020	0.012	0.047	NA	0.060	0.059
WY×10125132	–9.2834	1.47×10^{-6}	0.236	0.006	0.001	0.002	0.040	0.010	0.004	NA	0.000	0.037
mitoxantrone	–9.3439	1.32×10^{-6}	0.086	0.007	0.014	0.137	0.006	0.009	0.003	NA	0.063	0.022
AZD–8055	–9.4389	1.11×10^{-6}	0.004	0.005	0.013	0.016	0.249	0.012	0.004	NA	0.002	0.011
pirarubicin	–9.5076	9.78×10^{-7}	0.127	0.019	0.014	0.005	0.123	0.022	0.008	NA	0.007	0.007
PIK–75	–9.5689	8.73×10^{-7}	0.017	0.079	0.011	0.020	0.025	0.004	0.005	NA	0.047	0.123
bisindolylmaleimid×10ix	–9.5800	8.55×10^{-7}	0.019	0.074	0.019	0.028	0.013	0.007	0.010	NA	0.032	0.128
WZ–3105	–9.6105	8.12×10^{-7}	0.012	0.024	0.015	0.028	0.011	0.035	0.016	NA	0.078	NA
daunorubicin	–9.7609	6.16×10^{-7}	0.051	0.016	0.027	0.113	0.008	0.030	0.014	NA	0.014	0.043
XMD–1499	–9.9589	4.28×10^{-7}	0.031	0.060	0.114	0.010	0.016	0.006	0.002	NA	0.010	0.050
KU–0063794	–10.0240	3.79×10^{-7}	0.032	0.060	0.022	0.001	0.111	0.011	0.048	NA	0.003	0.007
aminopurvalanol–a	–10.0291	3.75×10^{-7}	0.079	0.007	0.084	0.040	0.013	0.017	0.048	NA	0.002	0.005
SU–11652	–10.0756	3.44×10^{-7}	0.007	0.020	0.065	0.007	NA	0.006	0.017	NA	0.047	0.021
5–iodotubercidin	–10.1309	3.11×10^{-7}	0.012	0.009	0.006	0.044	0.012	0.009	0.035	NA	0.061	NA
A–443644	–10.2476	2.52×10^{-7}	0.061	0.061	0.005	0.011	0.026	0.003	0.007	NA	0.007	NA
IKK–2–inhibitor–V	–10.5159	1.52×10^{-7}	0.010	0.017	0.036	0.003	0.020	0.023	0.006	NA	0.130	0.013
AS–601245	–10.7100	1.06×10^{-7}	0.013	0.058	0.027	0.039	0.034	0.050	0.007	NA	0.014	0.008
rottlerin	–10.8653	7.99×10^{-8}	0.013	0.052	0.010	0.002	0.005	0.033	0.020	0.076	0.065	0.072
BX–795	–11.0039	6.18×10^{-8}	0.002	0.014	0.066	0.028	0.011	0.080	0.011	NA	0.009	0.008
chromomycin–a3	–11.0634	5.51×10^{-8}	0.006	0.009	0.033	0.031	0.050	0.036	0.012	NA	0.033	0.017
niclosamide	–11.2932	3.58×10^{-8}	0.015	0.078	0.016	0.003	0.026	0.002	0.017	0.133	0.014	0.005

OSI–027	–11.4486	2.68×10^{-8}	0.019	0.016	0.039	0.018	0.011	0.036	0.044	NA	0.004	0.019
PF–562271	–11.4753	2.55×10^{-8}	0.016	0.034	0.011	0.006	0.016	0.011	0.026	NA	0.007	NA
doxorubicin	–11.5175	2.35×10^{-8}	0.044	0.001	0.011	0.089	0.006	0.005	0.003	NA	0.032	0.010
sirolimus	–12.4251	4.30×10^{-9}	0.007	0.011	0.029	0.010	0.001	0.003	0.007	0.147	0.018	0.001
TGX–115	–12.7582	2.33×10^{-9}	0.002	0.007	0.038	0.004	0.014	0.002	0.014	NA	0.045	0.020
PHA–793887	–12.7895	2.19×10^{-9}	0.024	0.004	0.017	0.010	0.021	0.012	0.009	NA	0.006	0.041
lestaurtinib	–14.8587	4.14×10^{-11}	0.005	0.003	0.004	0.041	0.010	0.003	0.001	NA	0.015	0.003
dorsomorphin	–16.0319	4.44×10^{-12}	0.002	0.003	0.009	0.003	0.013	0.004	0.001	NA	0.006	0.023

Table S5. The recurrent relationships which significantly upregulated expression by BI–2536 treatment for 6 hours

Gene|ID	REC	FDR	Relative rank									
			A375	A549	HA1E	HCC515	HEPG2	HT29	MCF7	NPC	PC3	VCAP
TERF2IP	16.2401	3.00×10^{-12}	0.998	0.991	0.999	1.000	0.978	0.997	1.000	NA	0.998	0.978
HBP1	15.1889	2.24×10^{-11}	0.987	0.994	0.998	0.997	0.981	0.999	0.988	NA	0.979	0.996
HIST1H2BK	14.8989	3.83×10^{-11}	0.997	0.972	0.998	0.990	0.975	0.985	0.999	NA	1.000	1.000
GADD45A	14.2400	1.38×10^{-10}	0.999	0.984	0.997	0.999	0.978	0.991	1.000	NA	1.000	0.953
NPC1	13.9453	2.44×10^{-10}	0.994	0.994	0.998	0.961	0.991	0.993	0.970	NA	0.999	0.992
WDR47	13.8236	3.05×10^{-10}	0.984	0.975	0.998	0.991	0.974	0.999	0.987	NA	0.981	0.999
CBX4	13.7357	3.61×10^{-10}	0.995	0.984	0.996	0.991	0.994	0.957	0.995	NA	0.993	0.979
KDM3A	13.7106	3.80×10^{-10}	1.000	1.000	0.979	1.000	0.992	1.000	0.924	NA	1.000	0.994
CIRBP	13.3293	7.90×10^{-10}	0.998	0.987	0.995	0.997	0.932	0.999	0.998	NA	0.998	0.970
H2BFS	13.1784	1.05×10^{-9}	0.989	0.957	0.999	0.974	0.972	0.986	0.997	NA	0.994	1.000
C18orf8	12.7507	2.36×10^{-9}	0.999	0.989	0.919	0.996	0.983	0.988	0.987	NA	0.994	0.998
PHKG2	12.7382	2.42×10^{-9}	0.992	0.985	0.988	0.966	0.972	1.000	0.989	NA	0.991	0.967
GTF2B	12.6226	2.98×10^{-9}	0.970	0.981	0.991	0.998	0.951	0.996	0.992	NA	0.989	0.981
HIST1H2AC	12.2542	5.93×10^{-9}	0.996	0.963	1.000	0.977	0.921	0.996	0.988	NA	0.998	0.996
DNAJB9	12.2188	6.34×10^{-9}	0.997	0.992	0.998	0.999	0.964	0.987	0.991	NA	0.932	0.973
HIST1H1C	11.9920	9.70×10^{-9}	0.991	0.916	0.994	0.967	0.975	0.987	0.999	NA	1.000	0.994
PNP	11.9450	1.06×10^{-8}	0.999	0.996	1.000	0.993	0.883	0.961	0.994	NA	0.999	1.000
HIST1H2BD	11.9048	1.14×10^{-8}	0.998	0.968	0.999	0.989	0.910	0.993	1.000	NA	0.974	0.990
HIST1H3H	11.9023	1.15×10^{-8}	0.983	0.900	0.998	0.979	0.978	1.000	0.992	NA	0.991	0.999

HIST1H2BH	11.7332	1.58×10^{-8}	0.970	0.939	0.999	0.995	0.937	0.999	0.994	NA	0.977	0.999
IDI1	11.4991	2.44×10^{-8}	0.997	0.994	0.993	0.986	0.939	0.982	0.985	NA	0.933	0.989
RSRP1	11.4039	2.91×10^{-8}	0.972	0.999	0.997	1.000	0.902	0.990	0.983	NA	0.991	0.961
DUSP14	11.2537	3.84×10^{-8}	0.999	1.000	0.995	0.996	0.944	0.993	0.979	NA	0.905	0.977
NXF1	11.1264	4.89×10^{-8}	0.956	0.987	0.997	0.992	0.889	0.990	0.995	NA	0.990	0.984
HMGCR	11.1155	4.99×10^{-8}	0.999	0.982	0.991	0.987	0.957	0.990	0.987	NA	0.887	0.999
ICAM5	11.0418	5.75×10^{-8}	0.954	0.988	0.994	0.984	0.998	0.993	0.974	NA	0.956	0.931
RYBP	11.0105	6.10×10^{-8}	0.985	0.985	0.981	0.981	0.882	0.988	0.981	NA	0.996	0.995
SNAP29	10.8684	7.95×10^{-8}	0.981	0.956	0.966	0.952	0.998	0.999	0.970	NA	0.945	0.994
SLC25A4	10.8512	8.19×10^{-8}	0.964	0.919	0.964	0.988	0.987	0.992	0.991	NA	0.991	0.965
SIRT1	10.8486	8.23×10^{-8}	0.957	0.974	0.987	0.969	0.947	0.992	0.968	NA	0.971	0.995
MAP1LC3B	10.7851	9.20×10^{-8}	0.999	0.953	0.981	0.982	0.908	0.978	0.994	NA	0.996	0.967
CDKN2AIP	10.7635	9.59×10^{-8}	0.980	0.975	0.992	0.930	0.989	0.975	0.973	NA	0.963	0.977
HECA	10.7484	9.87×10^{-8}	0.989	0.959	0.982	0.978	0.961	1.000	0.929	NA	0.961	0.996
RNF103	10.6514	1.17×10^{-7}	0.984	0.981	0.951	0.989	0.988	0.989	0.926	NA	0.993	0.947
FDFT1	10.5857	1.33×10^{-7}	0.994	0.941	0.976	0.971	0.960	0.987	0.949	NA	0.966	0.997
LPIN1	10.5034	1.55×10^{-7}	0.996	0.957	0.987	0.999	0.951	0.930	0.968	NA	0.957	0.993
HIST2H2BE	10.4753	1.63×10^{-7}	0.833	0.976	0.991	0.983	0.998	0.971	0.999	NA	0.994	0.999
ZNF654	10.4529	1.71×10^{-7}	0.990	0.984	0.892	0.968	1.000	0.995	0.951	NA	0.975	0.982
CDC34	10.3979	1.90×10^{-7}	0.999	0.989	0.984	0.985	0.967	0.871	0.999	NA	0.968	0.973
ALAS1	10.3738	1.99×10^{-7}	0.986	0.934	0.999	0.959	0.976	0.968	0.933	NA	0.999	0.975
CCNG2	10.3676	2.01×10^{-7}	0.996	0.993	0.996	0.998	0.933	0.977	0.954	NA	0.909	0.975
MOSPD1	10.3586	2.04×10^{-7}	0.993	0.969	0.980	0.997	0.887	0.939	0.991	NA	0.985	0.991

PLXNA3	10.2478	2.52×10^{-7}	0.953	0.956	0.986	0.977	0.961	0.933	0.981	NA	0.980	0.993
RRAGC	10.0364	3.71×10^{-7}	0.958	0.996	0.999	0.994	0.936	0.963	0.971	NA	0.979	0.910
RCOR1	9.8973	4.78×10^{-7}	0.988	0.960	0.993	0.960	0.876	0.957	0.965	NA	0.997	1.000
JUND	9.8370	5.34×10^{-7}	0.919	0.995	0.940	0.988	0.896	0.999	0.965	NA	0.993	0.997
CASP3	9.7923	5.81×10^{-7}	0.932	0.995	0.996	0.963	0.867	0.992	0.989	NA	0.957	1.000
CDK7	9.7474	6.32×10^{-7}	0.964	0.992	0.823	0.991	0.956	0.984	0.997	NA	0.998	0.987
CRK	9.7183	6.65×10^{-7}	0.994	0.893	0.997	0.976	0.964	0.963	0.936	NA	0.971	0.985
ALDOC	9.6805	7.12×10^{-7}	0.992	0.994	0.792	0.970	0.979	0.982	0.997	NA	0.997	0.989
SPSB3	9.6767	7.16×10^{-7}	0.981	0.906	0.947	0.975	0.949	0.936	0.995	NA	0.990	0.998
MAP3K3	9.6474	7.57×10^{-7}	0.997	0.964	0.997	0.919	0.989	0.994	0.886	NA	0.993	0.938
TMEM41B	9.6408	7.66×10^{-7}	0.996	0.908	0.925	0.957	0.968	0.971	0.994	NA	0.984	0.971
STXBP1	9.6058	8.19×10^{-7}	0.987	0.936	0.985	0.972	0.950	0.989	0.873	NA	0.995	0.985
CCNL2	9.5882	8.43×10^{-7}	0.939	0.966	0.990	0.991	0.824	0.985	0.996	NA	0.999	0.988
BTG2	9.5186	9.58×10^{-7}	0.971	0.968	0.959	0.959	0.986	0.947	0.967	NA	0.998	0.908
TOPORS	9.5054	9.82×10^{-7}	0.965	0.988	0.965	0.916	0.954	0.977	0.951	NA	0.992	0.951
MARCH2	9.4427	1.10×10^{-6}	0.979	0.990	0.953	0.980	0.918	0.894	0.992	NA	0.983	0.969
LGMN	9.4072	1.17×10^{-6}	0.982	0.921	1.000	0.998	0.848	0.988	0.999	NA	0.981	0.945
OSBP	9.3824	1.23×10^{-6}	0.994	0.942	0.990	0.973	0.913	0.935	0.997	NA	0.985	0.922
GSDMB	9.3778	1.24×10^{-6}	0.993	0.966	0.957	0.877	0.911	0.981	0.986	NA	0.988	0.995
CSRNP2	9.3661	1.26×10^{-6}	0.988	0.962	0.990	0.875	0.928	0.990	0.970	NA	0.951	0.998
TNFSF9	9.3124	1.40×10^{-6}	0.965	0.976	0.849	0.954	0.982	0.991	0.994	NA	0.973	0.965
SOX3	9.2973	1.43×10^{-6}	0.928	0.980	0.997	0.958	0.971	0.959	0.883	NA	0.985	0.983
ZNF394	9.2765	1.49×10^{-6}	0.967	0.948	0.961	0.986	0.948	0.981	0.993	NA	0.973	0.886

YPEL5	9.2725	1.50×10^{-6}	0.963	0.963	0.897	0.966	0.963	0.998	0.998	NA	0.907	0.987
PRDM1	9.2549	1.55×10^{-6}	0.920	0.971	0.993	0.976	0.995	0.974	0.982	NA	0.997	0.837
GAS6	9.2540	1.55×10^{-6}	0.880	0.999	0.994	0.970	0.969	0.962	0.995	NA	0.988	0.886
GUCA1A	9.2496	1.57×10^{-6}	0.839	0.977	0.996	0.963	0.970	0.978	0.955	NA	0.982	0.984
TPST2	9.2406	1.59×10^{-6}	0.990	0.952	0.912	0.946	0.977	0.902	0.984	NA	0.989	0.985
KLHL24	9.2308	1.62×10^{-6}	0.966	0.966	0.929	0.971	0.962	0.987	0.871	NA	0.995	0.992
TAF12	9.2290	1.63×10^{-6}	0.925	0.910	0.985	0.975	0.985	0.899	0.994	NA	0.986	0.978
SNRK	9.2238	1.64×10^{-6}	0.993	0.943	0.995	0.977	0.967	0.973	0.834	NA	0.968	0.993
TM7SF2	9.2044	1.70×10^{-6}	0.952	0.933	0.999	0.986	0.840	0.994	0.997	NA	0.976	0.964
NEU1	9.1666	1.82×10^{-6}	0.989	0.998	0.999	0.996	0.770	0.983	0.989	NA	0.969	0.957
BNIP3L	9.1302	1.94×10^{-6}	1.000	1.000	0.996	0.982	0.849	0.992	0.932	NA	0.936	0.946
HERPUD1	9.1008	2.05×10^{-6}	0.997	0.998	0.995	0.998	0.954	0.979	0.917	NA	0.802	0.999
CDKN2D	9.0915	2.08×10^{-6}	0.986	0.964	0.864	0.978	0.906	0.981	0.982	NA	0.998	0.967
CCNL1	9.0627	2.20×10^{-6}	0.985	0.982	0.964	0.931	0.940	0.944	0.971	NA	0.980	0.920
ABHD3	9.0491	2.25×10^{-6}	0.993	0.976	0.993	0.988	0.746	0.997	0.983	NA	0.984	0.985
ZBTB43	9.0132	2.41×10^{-6}	0.949	0.895	0.912	0.953	0.984	0.998	0.986	NA	0.983	0.956
DNAJB4	9.0081	2.43×10^{-6}	0.983	0.915	0.974	0.935	0.897	0.988	0.964	NA	0.990	0.969
HIST1H2BO	8.9768	2.57×10^{-6}	0.862	0.945	0.996	0.858	0.997	0.996	0.999	NA	0.977	0.991
CCDC92	8.9699	2.60×10^{-6}	0.994	0.947	0.968	0.998	0.9^{6}	0.954	0.946	NA	0.997	0.899
TMEM57	8.9189	2.85×10^{-6}	0.926	0.959	0.976	0.959	0.982	0.953	0.964	NA	0.971	0.911
DESI2	8.9097	2.91×10^{-6}	0.990	0.993	0.857	0.975	0.974	0.975	0.907	NA	0.958	0.980
COPE	8.8846	3.04×10^{-6}	0.905	0.927	0.978	0.954	0.995	0.974	0.990	NA	0.933	0.945
ZFP36	8.8759	3.09×10^{-6}	0.972	0.957	1.000	0.974	0.973	0.843	0.925	NA	0.999	0.964

SSR4	8.8455	3.26×10^{-6}	0.811	0.954	0.992	0.982	0.950	0.981	0.992	NA	0.956	0.990
DAZL	8.8434	3.28×10^{-6}	0.877	0.986	0.989	0.956	0.892	0.944	0.998	NA	0.977	0.983
SQLE	8.8405	3.29×10^{-6}	0.975	0.994	0.983	0.967	0.896	0.989	0.983	NA	0.872	0.940
TKTL1	8.8354	3.33×10^{-6}	0.918	0.939	0.965	0.937	0.992	0.931	0.975	NA	0.952	0.986
NOSIP	8.8174	3.44×10^{-6}	0.906	0.994	0.996	0.926	0.864	0.999	0.993	NA	0.927	0.996
NTAN1	8.8115	3.47×10^{-6}	0.979	0.939	0.992	0.915	0.862	1.000	0.950	NA	0.996	0.966
NRBP1	8.7694	3.75×10^{-6}	0.955	0.982	0.968	0.961	0.975	0.995	0.927	NA	0.955	0.874
ULK1	8.7458	3.92×10^{-6}	0.993	0.873	0.989	0.985	0.937	0.986	0.943	NA	0.925	0.960
BSG	8.7405	3.95×10^{-6}	0.909	0.863	0.969	0.982	0.953	0.991	0.999	NA	0.968	0.956
AKR7A3	8.7188	4.11×10^{-6}	0.926	0.985	0.935	0.881	0.979	0.982	0.943	NA	0.999	0.954
MGEA5	8.7156	4.14×10^{-6}	0.988	0.989	0.993	0.950	0.924	0.997	0.989	NA	0.852	0.908
IER5	8.6747	4.46×10^{-6}	0.974	0.912	0.980	0.954	0.839	0.981	0.990	NA	0.995	0.960
ASMTL	8.6611	4.57×10^{-6}	0.972	0.974	0.976	0.985	0.821	0.983	0.990	NA	0.967	0.918
FGD6	8.6278	4.86×10^{-6}	0.987	0.929	0.994	0.900	0.996	0.846	0.946	NA	0.988	0.995
PRKG1	8.6149	4.98×10^{-6}	0.943	0.954	0.968	0.936	0.981	0.860	0.992	NA	0.965	0.976
MICAL1	8.6116	5.01×10^{-6}	0.982	0.932	0.979	1.000	0.811	0.974	0.970	NA	0.944	0.990
HIST3H2A	8.6088	5.03×10^{-6}	0.946	0.997	0.894	0.946	0.985	0.994	0.984	NA	0.887	0.941
MYO1D	8.6087	5.03×10^{-6}	0.935	0.959	0.967	0.930	0.993	0.964	0.966	NA	0.904	0.952
HLA–G	8.5964	5.15×10^{-6}	0.935	0.957	0.977	0.990	0.828	0.991	0.985	NA	0.947	0.968
TUFT1	8.5910	5.20×10^{-6}	0.982	0.859	0.926	0.960	0.953	0.961	0.974	NA	0.992	0.964
BRD2	8.5423	5.67×10^{-6}	0.992	0.982	0.984	0.995	0.800	0.991	0.991	NA	0.866	0.982
BRAP	8.4425	6.81×10^{-6}	0.972	0.995	0.990	0.978	0.885	0.926	0.997	NA	0.980	0.839
ELOVL4	8.4292	6.97×10^{-6}	0.890	0.959	0.985	0.997	0.917	0.971	0.985	NA	0.886	0.965

GADD45B	8.4046	7.30×10^{-6}	0.991	0.993	0.999	0.834	0.970	0.916	0.911	NA	1.000	0.944
KIAA1033	8.4032	7.31×10^{-6}	0.907	0.937	0.818	0.990	0.992	0.989	0.968	NA	0.974	0.983
AKR7A2	8.3797	7.64×10^{-6}	0.923	0.986	0.997	0.966	0.736	0.970	0.999	NA	0.999	0.998
GABARAPL2	8.3707	7.75×10^{-6}	0.991	0.976	0.918	0.957	0.877	0.975	0.976	NA	0.921	0.953
MTM1	8.3703	7.76×10^{-6}	0.926	0.970	0.999	0.966	0.872	0.967	0.930	NA	0.978	0.937
SMC6	8.3667	7.81×10^{-6}	0.885	0.932	0.931	0.997	0.973	0.967	0.981	NA	0.986	0.893
CDKN1A	8.3665	7.81×10^{-6}	0.999	0.988	0.979	0.948	0.889	0.985	0.959	NA	0.996	0.813
FAM53C	8.3532	8.01×10^{-6}	0.991	0.958	0.874	0.987	0.905	0.979	0.931	NA	0.992	0.928
INSIG1	8.3244	8.45×10^{-6}	0.999	0.997	1.000	0.988	0.646	0.991	0.996	NA	0.999	0.990
KIAA0513	8.3209	8.50×10^{-6}	0.971	0.932	0.960	0.981	0.995	0.991	0.894	NA	0.982	0.839
TST	8.2848	9.07×10^{-6}	0.973	0.890	0.988	0.964	0.993	0.924	0.988	NA	0.987	0.835
RBM6	8.2823	9.11×10^{-6}	0.947	0.862	0.846	0.984	0.986	0.954	1.000	NA	0.983	0.980
MSMO1	8.2821	9.12×10^{-6}	0.993	0.987	0.998	0.973	0.762	0.990	0.995	NA	0.938	0.921
SREBF2	8.2083	1.04×10^{-5}	0.984	0.952	0.995	0.916	0.920	0.967	0.908	NA	0.919	0.963
ZNF10	8.2019	1.05×10^{-5}	0.975	0.956	0.998	0.968	0.801	0.973	0.904	NA	0.963	0.998
BNIP3	8.1718	1.11×10^{-5}	0.997	0.985	0.998	0.992	0.901	0.768	0.987	NA	0.985	0.928
WIPI1	8.1474	1.16×10^{-5}	0.966	0.968	0.895	0.985	0.949	0.997	0.817	NA	0.992	0.957
MTMR14	8.0993	1.27×10^{-5}	0.960	0.978	0.973	0.993	0.999	0.844	0.928	NA	0.881	0.963
PI4KA	8.0819	1.31×10^{-5}	0.986	0.883	0.968	0.996	0.896	0.992	0.954	NA	0.916	0.921
NAA10	8.0655	1.35×10^{-5}	0.965	0.971	0.974	0.880	0.990	0.919	0.891	NA	0.995	0.925
UBE2A	8.0554	1.37×10^{-5}	1.000	0.988	0.986	0.771	0.979	0.982	0.932	NA	0.997	0.892
NRBF2	8.0451	1.40×10^{-5}	0.963	0.970	0.991	0.989	0.756	0.976	0.968	NA	0.990	0.924
SLC9A6	8.0420	1.40×10^{-5}	0.915	0.934	0.970	0.949	0.943	0.956	0.896	NA	0.986	0.952

HIST1H2AM	8.0210	1.46×10^{-5}	0.946	0.917	0.965	0.927	0.996	0.995	0.878	NA	0.891	0.989
EAF2	7.9897	1.54×10^{-5}	0.999	0.926	0.828	0.990	0.988	0.851	0.961	NA	0.975	0.993
ING1	7.9619	1.63×10^{-5}	0.932	0.999	0.991	0.973	0.815	0.955	0.979	NA	0.996	0.866
IP6K2	7.9401	1.69×10^{-5}	0.976	0.999	0.926	0.954	0.836	0.987	0.975	NA	0.922	0.921
TMEM66	7.9247	1.74×10^{-5}	0.967	0.958	0.976	0.970	0.852	0.997	0.942	NA	0.930	0.899
DNM1L	7.9116	1.78×10^{-5}	0.979	0.927	0.931	0.950	0.944	0.999	0.865	NA	0.936	0.956
MAGEB2	7.8556	1.97×10^{-5}	0.941	0.988	0.944	0.977	0.916	0.800	0.976	NA	0.984	0.962
NDUFA2	7.8244	2.08×10^{-5}	0.983	0.924	0.987	0.991	0.965	0.903	0.973	NA	0.933	0.823
CCDC93	7.8005	2.17×10^{-5}	0.982	0.999	0.994	0.975	0.947	0.864	0.938	NA	0.906	0.870
NARF	7.7904	2.21×10^{-5}	0.961	0.998	0.989	0.955	0.848	0.953	0.974	NA	0.871	0.925
TCEB2	7.7892	2.22×10^{-5}	0.952	0.965	0.982	0.935	0.978	0.929	0.987	NA	0.853	0.892
HEXIM1	7.7837	2.24×10^{-5}	0.894	0.918	0.965	0.989	0.955	0.992	0.974	NA	0.967	0.821
B3GALT2	7.7827	2.24×10^{-5}	0.881	0.968	0.842	0.984	0.945	0.962	0.971	NA	0.965	0.955
MVD	7.7672	2.31×10^{-5}	0.984	0.987	0.995	0.977	0.889	0.968	0.936	NA	0.816	0.923
DAAM1	7.7440	2.40×10^{-5}	0.951	0.999	0.869	0.891	0.939	0.999	0.982	NA	0.860	0.982
RHCE	7.7381	2.43×10^{-5}	0.936	0.812	0.942	0.970	0.955	0.948	0.994	NA	0.939	0.973
HAUS3	7.7228	2.49×10^{-5}	0.956	0.946	0.988	0.969	0.970	0.932	0.853	NA	0.897	0.950
BTG1	7.7101	2.55×10^{-5}	0.956	0.936	0.904	0.931	0.882	0.995	0.947	NA	0.927	0.980
TRIM36	7.6987	2.60×10^{-5}	0.761	0.926	0.975	0.990	0.971	0.907	0.996	NA	0.997	0.953
CD9	7.6942	2.62×10^{-5}	0.978	0.964	0.952	0.968	0.949	0.865	0.868	NA	0.944	0.969
SNX16	7.6891	2.65×10^{-5}	0.855	0.984	0.948	0.963	0.893	0.979	0.859	NA	0.991	0.991
NFKBIE	7.6712	2.74×10^{-5}	0.987	0.951	0.898	0.783	0.995	0.955	0.927	NA	0.975	0.997
ARHGEF7	7.6677	2.75×10^{-5}	0.929	0.999	0.987	0.942	0.944	0.830	0.949	NA	0.890	0.988

SIRT2	7.6648	2.77×10^{-5}	0.917	0.982	0.950	0.914	0.979	0.966	0.923	NA	0.967	0.856
RSRC2	7.6383	2.90×10^{-5}	0.987	0.913	0.996	0.994	0.872	0.963	0.968	NA	0.845	0.917
AHDC1	7.6336	2.93×10^{-5}	0.990	0.781	0.952	0.924	0.980	0.925	0.980	NA	0.943	0.986
PI4K2A	7.6246	2.97×10^{-5}	0.846	0.995	0.983	0.956	0.822	0.960	0.981	NA	0.975	0.938
RBBP6	7.6226	2.98×10^{-5}	0.975	0.956	0.820	0.995	0.886	0.981	0.972	NA	0.971	0.898
DMTF1	7.6167	3.02×10^{-5}	0.993	0.959	0.902	0.974	0.962	0.888	0.800	NA	0.980	0.998
AZIN1	7.5691	3.28×10^{-5}	0.886	0.902	0.951	0.948	0.968	0.959	0.952	NA	0.916	0.952
GDI1	7.5321	3.51×10^{-5}	0.975	0.898	0.994	0.856	0.960	0.955	0.956	NA	0.847	0.998
ATP6V1G1	7.5164	3.61×10^{-5}	0.949	0.961	0.983	0.990	0.732	0.995	0.885	NA	0.984	0.980
AKAP17A	7.4719	3.90×10^{-5}	0.956	0.926	0.971	0.848	0.996	0.863	0.939	NA	0.959	0.969
LIPG	7.4619	3.97×10^{-5}	0.930	0.936	0.941	0.999	0.763	0.943	0.999	NA	0.937	0.992
CALCOCO1	7.4488	4.06×10^{-5}	0.866	0.858	0.997	0.993	0.904	0.958	0.990	NA	0.931	0.929
ZNF268	7.4210	4.27×10^{-5}	0.952	0.968	0.978	0.995	0.971	0.798	0.900	NA	0.948	0.914
PAFAH2	7.4068	4.38×10^{-5}	0.8007	0.982	0.993	0.920	0.952	0.836	0.976	NA	0.982	0.982
ATPIF1	7.4009	4.42×10^{-5}	0.939	0.980	0.903	0.997	0.774	0.985	0.959	NA	0.963	0.927
FOXO3	7.3958	4.47×10^{-5}	0.934	0.788	0.831	0.983	0.918	0.996	0.998	NA	0.994	0.992
SAT1	7.3722	4.66×10^{-5}	0.954	0.972	0.996	0.932	0.826	0.926	0.972	NA	0.911	0.924
AKIRIN1	7.3713	4.66×10^{-5}	0.993	0.957	0.921	0.940	0.843	0.984	0.948	NA	0.991	0.838
SDC3	7.3464	4.87×10^{-5}	0.969	0.941	0.788	0.895	0.989	0.915	0.962	NA	0.971	0.987
BLK	7.3320	5.00×10^{-5}	0.918	0.956	0.927	0.867	0.866	0.987	0.991	NA	0.946	0.947
MAN2A1	7.3073	5.22×10^{-5}	0.867	0.857	0.971	0.797	0.956	0.993	0.994	NA	0.994	0.987
FADS3	7.2913	5.37×10^{-5}	0.985	0.921	0.913	0.950	0.917	0.985	0.843	NA	0.895	0.990
TRNAU1AP	7.2819	5.46×10^{-5}	0.995	0.998	0.9^{7}	0.800	0.997	0.863	0.917	NA	0.969	0.963

CAP1	7.2554	5.73×10^{-5}	0.982	0.960	0.750	0.922	0.867	0.997	0.954	NA	0.997	0.985
PLEKHF2	7.2418	5.87×10^{-5}	0.957	0.985	0.958	0.914	0.965	0.967	0.872	NA	0.811	0.965
TXNIP	7.2057	6.25×10^{-5}	0.910	0.958	0.982	0.958	0.954	0.797	0.962	NA	0.968	0.902
CDC37L1	7.2037	6.27×10^{-5}	0.981	0.978	0.978	0.847	0.821	0.958	0.988	NA	0.994	0.852
VAMP1	7.1987	6.32×10^{-5}	0.974	0.946	0.888	0.839	0.998	0.876	0.957	NA	0.974	0.935
COX6A1	7.1799	6.54×10^{-5}	0.984	0.997	0.950	0.969	0.738	0.952	0.993	NA	0.959	0.861
ATP2A3	7.1751	6.60×10^{-5}	0.961	0.995	0.965	0.859	0.991	0.850	0.888	NA	0.960	0.915
DHCR7	7.1741	6.61×10^{-5}	0.944	0.981	0.870	0.960	0.710	0.977	0.988	NA	0.988	0.994
CBFA2T3	7.1694	6.67×10^{-5}	0.953	0.991	0.977	0.949	0.977	0.879	0.875	NA	0.813	0.972
RIPK1	7.1575	6.81×10^{-5}	0.993	0.989	0.924	0.813	0.985	0.790	0.981	NA	0.962	0.957
ZNF223	7.1433	6.99×10^{-5}	0.757	0.952	0.963	0.957	0.913	0.910	0.976	NA	0.990	0.971
PMAIP1	7.1198	7.29×10^{-5}	0.992	0.987	0.999	0.999	0.927	0.996	0.774	NA	0.985	0.749
CAAP1	7.1108	7.41×10^{-5}	0.997	0.917	0.986	0.883	0.894	0.793	0.983	NA	0.945	0.980
JUNB	6.9943	9.10×10^{-5}	0.959	0.958	0.898	0.876	0.888	0.992	0.884	NA	0.980	0.913
IFRD1	6.9879	9.20×10^{-5}	0.998	0.962	0.998	0.983	0.683	0.980	0.951	NA	0.846	0.992
PINK1	6.9872	9.21×10^{-5}	0.826	0.908	0.938	0.995	0.865	0.967	0.982	NA	0.917	0.953
SLC26A6	6.9755	9.41×10^{-5}	0.730	0.990	0.864	0.923	0.993	0.989	0.963	NA	0.937	0.981
DSTN	6.9639	9.60×10^{-5}	0.973	0.875	0.883	0.989	0.938	0.978	0.9044	NA	0.924	0.878
ANKRD46	6.9480	9.87×10^{-5}	0.988	0.930	0.914	0.804	0.957	0.917	0.977	NA	0.972	0.886
SERTAD3	6.9416	9.98×10^{-5}	0.979	0.984	0.949	0.829	0.951	0.953	0.756	NA	0.986	0.973
KLF13	6.9193	1.04×10^{-4}	0.864	0.851	0.942	0.991	0.973	0.811	0.993	NA	0.990	0.932
DCAF10	6.9079	1.06×10^{-4}	0.996	0.921	0.956	0.965	0.970	0.811	0.984	NA	0.902	0.837
MARCH3	6.8646	1.15×10^{-4}	0.933	0.889	0.961	0.817	0.839	0.944	0.993	NA	0.971	0.986

ZNF211	6.8564	1.16×10^{-4}	0.907	0.992	0.988	0.954	0.869	0.869	0.997	NA	0.953	0.805
FDPS	6.8496	1.18×10^{-4}	0.900	0.976	0.993	0.981	0.803	0.967	0.978	NA	0.796	0.948
PIKFYVE	6.8405	1.19×10^{-4}	0.971	0.890	0.975	0.964	0.868	0.937	0.935	NA	0.858	0.922
CTH	6.8290	1.22×10^{-4}	0.994	0.997	0.989	0.989	0.534	0.983	0.994	NA	0.981	0.981
HDAC5	6.8008	1.28×10^{-4}	0.797	0.975	0.951	0.948	0.927	0.962	0.938	NA	0.884	0.935
NDUFS7	6.7838	1.32×10^{-4}	0.808	0.967	0.925	0.982	0.945	0.926	0.993	NA	0.921	0.850
MTERFD1	6.7743	1.35×10^{-4}	0.994	0.997	0.895	0.983	0.998	0.795	0.942	NA	0.829	0.890
POU5F1P4	6.7701	1.36×10^{-4}	0.924	0.836	0.980	0.919	0.927	0.980	0.980	NA	0.826	0.943
AP3S1	6.7566	1.39×10^{-4}	0.960	0.935	0.760	0.992	0.939	0.952	0.907	NA	0.946	0.925
KCTD13	6.7357	1.44×10^{-4}	0.986	0.820	0.982	0.907	0.948	0.841	0.981	NA	0.875	0.970
ZNF124	6.7355	1.44×10^{-4}	0.944	0.854	0.956	0.931	0.873	0.983	0.936	NA	0.938	0.885
H2AFV	6.7241	1.47×10^{-4}	0.974	0.956	0.839	0.964	0.983	0.863	0.940	NA	0.882	0.901
ATP6V0D1	6.7200	1.48×10^{-4}	0.991	0.962	0.861	0.935	0.857	0.905	0.963	NA	0.912	0.911
GNRH1	6.7186	1.48×10^{-4}	0.845	0.975	0.919	0.963	0.930	0.977	0.977	NA	0.977	0.752
LSS	6.7003	1.53×10^{-4}	0.970	0.997	0.908	0.991	0.748	0.935	0.985	NA	0.803	0.986
DDIT3	6.6897	1.56×10^{-4}	0.879	0.994	0.995	0.999	0.775	0.843	0.887	NA	0.970	0.969
RIOK3	6.6770	1.60×10^{-4}	0.968	0.828	0.838	0.964	0.948	0.990	0.924	NA	0.931	0.905
CDH13	6.6469	1.68×10^{-4}	0.905	0.984	0.884	0.909	0.929	0.923	0.992	NA	0.989	0.781
RLF	6.6442	1.69×10^{-4}	0.991	0.971	0.961	0.987	0.990	0.846	0.895	NA	0.961	0.715
LIN37	6.6438	1.69×10^{-4}	0.887	0.979	0.978	0.947	0.977	0.893	0.973	NA	0.786	0.876
HIST1H1T	6.6381	1.71×10^{-4}	0.916	0.877	0.973	0.928	0.997	0.998	0.789	NA	0.911	0.905
TUBB2A	6.6378	1.71×10^{-4}	0.887	0.994	0.765	0.932	0.915	0.932	0.986	NA	0.921	0.963
MSTN	6.6303	1.73×10^{-4}	0.801	0.922	0.977	0.988	0.858	0.929	0.984	NA	0.889	0.942

PIM2	6.6119	1.79×10^{-4}	0.890	0.909	0.951	0.971	0.907	0.844	0.953	NA	0.966	0.886
ATG2B	6.6085	1.80×10^{-4}	0.852	0.893	0.991	0.992	0.799	0.995	0.966	NA	0.959	0.846
WDR44	6.6009	1.83×10^{-4}	0.959	0.979	0.956	0.944	0.929	0.800	0.911	NA	0.965	0.840
NOTCH1	6.6006	1.83×10^{-4}	0.990	0.999	0.995	0.971	0.565	0.989	0.881	NA	0.992	0.996
DYNC1H1	6.5793	1.90×10^{-4}	0.940	0.924	0.915	0.954	0.839	0.966	0.894	NA	0.912	0.926
FAM50A	6.5753	1.91×10^{-4}	0.934	0.990	0.926	0.744	0.987	0.966	0.946	NA	0.854	0.943
RUNDC3B	6.5613	1.96×10^{-4}	0.934	0.991	0.832	0.911	0.815	0.996	0.861	NA	0.993	0.948
TUBB3	6.5575	1.97×10^{-4}	0.974	0.967	0.658	0.930	0.896	0.952	0.995	NA	0.955	0.986
HLA–E	6.5557	1.98×10^{-4}	0.998	0.993	0.990	0.997	0.592	0.946	0.985	NA	0.937	0.912
NPPC	6.5544	1.98×10^{-4}	0.792	0.835	0.974	0.926	0.925	0.977	0.942	NA	0.946	0.959
GNAI2	6.5431	2.02×10^{-4}	1.000	0.804	0.993	0.966	0.808	0.999	0.866	NA	0.995	0.858
IFI6	6.5374	2.04×10^{-4}	0.942	0.967	0.963	0.901	0.834	0.942	0.927	NA	0.949	0.840
CDC37	6.5366	2.05×10^{-4}	0.705	0.920	0.957	0.984	0.988	0.865	0.989	NA	0.992	0.895
NTRK1	6.5224	2.10×10^{-4}	0.829	0.975	0.852	0.781	0.947	0.972	0.995	NA	0.960	0.969
GUK1	6.5175	2.12×10^{-4}	0.989	0.986	0.821	0.909	0.822	0.988	0.981	NA	0.899	0.876
ANKRD10	6.4986	2.19×10^{-4}	0.998	0.996	0.930	0.827	0.867	0.937	0.999	NA	0.814	0.900
BLZF1	6.4949	2.20×10^{-4}	0.859	0.995	0.956	0.798	0.940	0.964	0.925	NA	0.964	0.863
ABCA7	6.4868	2.23×10^{-4}	0.990	0.992	0.965	0.991	0.620	0.870	0.984	NA	0.911	0.999
TSPYL2	6.4653	2.32×10^{-4}	0.981	0.899	0.989	0.951	0.973	0.962	0.786	NA	0.828	0.894
CPT2	6.4597	2.34×10^{-4}	0.818	0.987	0.922	0.933	0.981	0.983	0.965	NA	0.988	0.706
TPP1	6.4587	2.34×10^{-4}	0.848	0.868	0.963	0.910	0.947	0.876	0.981	NA	0.953	0.901
RNF38	6.4473	2.39×10^{-4}	0.953	0.892	0.980	0.986	0.928	0.914	0.740	NA	0.906	0.962
ZCCHC6	6.4284	2.47×10^{-4}	0.954	0.938	0.855	0.985	0.895	0.935	0.935	NA	0.935	0.811

ABHD17A	6.4158	2.53×10^{-4}	0.976	0.915	0.873	0.967	0.837	0.794	0.984	NA	0.967	0.937
KLK5	6.4076	2.56×10^{-4}	0.937	0.886	0.941	0.903	0.859	0.934	0.980	NA	0.908	0.885
ROGDI	6.3892	2.65×10^{-4}	0.921	0.697	0.972	0.942	0.997	0.909	0.991	NA	0.937	0.898
ZNF331	6.3830	2.68×10^{-4}	0.984	0.891	0.981	0.801	0.844	0.933	0.959	NA	0.889	0.959
STARD5	6.3758	2.71×10^{-4}	0.917	0.933	0.910	0.934	0.959	0.905	0.908	NA	0.978	0.790
CYP46A1	6.3737	2.72×10^{-4}	0.945	0.867	0.915	0.879	0.948	0.955	0.896	NA	0.876	0.944
HSPA6	6.3712	2.73×10^{-4}	0.916	0.954	0.973	0.983	0.704	0.954	0.862	NA	0.996	0.918
ATP6V0A1	6.3627	2.77×10^{-4}	0.971	0.905	0.932	0.941	0.802	0.859	0.968	NA	0.932	0.921
DYNC1LI1	6.3549	2.81×10^{-4}	0.997	0.979	0.985	0.969	0.936	0.948	0.842	NA	0.860	0.735
PPP1R15A	6.3534	2.82×10^{-4}	0.911	0.894	0.975	0.978	0.673	0.933	0.993	NA	0.942	0.965
KDM7A	6.3390	2.89×10^{-4}	0.944	0.954	0.963	0.977	0.889	0.873	0.953	NA	0.873	0.803
ST6GALNAC5	6.3298	2.94×10^{-4}	0.834	0.943	0.987	0.813	0.949	0.892	0.988	NA	0.943	0.879
ARSA	6.3161	3.01×10^{-4}	0.851	0.954	0.997	0.973	0.971	0.938	0.978	NA	0.980	0.635
SLC30A1	6.2963	3.11×10^{-4}	0.989	0.937	0.810	0.962	0.896	0.826	0.927	NA	0.925	0.949
TNNI3	6.2776	3.22×10^{-4}	0.948	0.689	0.970	0.941	0.990	0.796	0.998	NA	0.963	0.958
CTSK	6.2717	3.25×10^{-4}	0.926	0.984	0.892	0.976	0.767	0.972	0.886	NA	0.994	0.830
HADHB	6.2409	3.43×10^{-4}	0.930	0.928	0.868	0.914	0.876	0.994	0.873	NA	0.856	0.962
C12orf5	6.2238	3.54×10^{-4}	0.946	0.965	0.992	0.701	0.856	0.848	0.957	NA	0.983	0.987
NR4A3	6.2231	3.54×10^{-4}	0.976	0.969	0.961	0.851	0.873	0.972	0.767	NA	0.973	0.872
SQSTM1	6.2022	3.67×10^{-4}	0.810	0.990	1.000	0.963	0.865	0.919	0.757	NA	0.923	0.992
DNAJC6	6.1788	3.82×10^{-4}	0.482	0.989	0.974	0.999	0.985	0.963	0.993	NA	0.970	0.997
STK3	6.1732	3.86×10^{-4}	0.784	0.888	0.959	0.979	0.980	0.829	0.890	NA	0.941	0.949
CPSF3L	6.1715	3.87×10^{-4}	0.948	0.874	0.978	0.993	0.676	0.872	1.000	NA	0.995	0.893

ERO1LB	6.1630	$3.93×10^{-4}$	0.650	0.968	0.995	0.983	0.858	0.930	0.961	NA	0.984	0.906
FOXN3	6.1569	$3.97×10^{-4}$	0.734	0.908	0.909	0.997	0.737	0.998	0.984	NA	0.979	0.982
PELI1	6.1507	$4.01×10^{-4}$	0.831	0.914	0.976	0.955	0.757	0.967	0.979	NA	0.948	0.872
VAMP3	6.1504	$4.01×10^{-4}$	0.934	0.896	0.908	0.963	0.913	0.749	0.974	NA	0.912	0.946
APBB3	6.1354	$4.12×10^{-4}$	0.900	0.896	0.983	0.947	0.831	0.935	0.860	NA	0.907	0.920
ZBTB5	6.1275	$4.18×10^{-4}$	0.977	0.966	0.777	0.949	0.825	0.965	0.906	NA	0.872	0.955
FGL2	6.1274	$4.18×10^{-4}$	0.883	0.930	0.967	0.898	0.910	0.834	0.977	NA	0.892	0.883
YIPF3	6.1223	$4.22×10^{-4}$	0.845	0.798	0.925	0.913	0.974	0.948	0.988	NA	0.888	0.903
SS18L1	6.0974	$4.40×10^{-4}$	0.966	0.799	0.968	0.950	0.923	0.896	0.889	NA	0.968	0.820
HSD17B12	6.0879	$4.47×10^{-4}$	0.776	0.990	0.928	0.984	0.676	0.963	0.991	NA	0.957	0.954
DNAJB6	6.0777	$4.55×10^{-4}$	0.958	0.973	0.710	0.902	0.945	0.979	0.893	NA	0.948	0.882
IGF2R	6.0642	$4.66×10^{-4}$	0.899	0.873	0.842	0.970	0.674	0.984	0.988	NA	0.995	0.984
HLA–F	6.0576	$4.71×10^{-4}$	0.836	0.965	0.971	0.945	0.834	0.801	0.964	NA	0.882	0.976
NNAT	6.0423	$4.84×10^{-4}$	0.761	0.861	0.946	0.933	0.931	0.990	0.907	NA	0.867	0.975
BCL10	6.0296	$4.95×10^{-4}$	0.970	0.923	0.936	0.834	0.909	0.957	0.782	NA	0.931	0.921
INSR	6.0235	$5.00×10^{-4}$	0.917	0.859	0.892	0.916	0.965	0.827	0.925	NA	0.984	0.871
ATP6V1D	5.9886	$5.31×10^{-4}$	0.941	0.638	0.934	0.962	0.938	0.996	0.938	NA	0.943	0.906
FEM1C	5.9869	$5.33×10^{-4}$	0.940	0.991	0.945	0.910	0.900	0.772	0.941	NA	0.779	0.986
PSAP	5.9868	$5.33×10^{-4}$	0.853	0.924	0.982	0.995	0.713	0.959	0.995	NA	0.908	0.847
NR5A2	5.9868	$5.33×10^{-4}$	0.964	0.909	0.924	0.765	0.959	0.929	0.838	NA	0.994	0.876
TOB2	5.9832	$5.36×10^{-4}$	0.922	0.900	0.873	0.728	0.928	0.956	0.909	NA	0.969	0.977
PTTG1IP	5.9551	$5.62×10^{-4}$	0.958	0.958	0.931	0.924	0.677	0.951	0.902	NA	0.878	0.992
NAB2	5.9539	$5.64×10^{-4}$	0.979	0.955	0.937	0.876	0.998	0.952	0.611	NA	0.918	0.978

CCDC121	5.9537	5.64×10^{-4}	0.763	0.987	0.955	0.935	0.970	0.885	0.928	NA	0.968	0.771
WDR78	5.9447	5.73×10^{-4}	0.920	0.904	0.865	0.946	0.832	0.855	0.990	NA	0.885	0.938
GAL3ST4	5.9431	5.74×10^{-4}	0.635	0.930	0.971	0.973	0.854	0.993	0.942	NA	0.915	0.977
ANKRD11	5.9370	5.80×10^{-4}	0.995	0.858	0.837	0.784	0.961	0.975	0.886	NA	0.969	0.883
SSBP3	5.9337	5.84×10^{-4}	0.862	0.730	0.956	0.857	0.978	0.973	0.976	NA	0.866	0.960
EGR4	5.9268	5.91×10^{-4}	0.890	0.983	0.751	0.990	0.773	0.922	0.890	NA	0.978	0.983
TIPARP	5.9017	6.17×10^{-4}	0.881	0.993	0.999	0.753	0.993	0.615	0.996	NA	0.991	0.995
EPHX1	5.8957	6.24×10^{-4}	0.995	0.828	0.936	0.983	0.688	0.956	0.953	NA	0.918	0.903
GOLGB1	5.8940	6.26×10^{-4}	0.819	0.991	0.906	0.982	0.991	0.913	0.891	NA	0.772	0.876
SUGP1	5.8895	6.31×10^{-4}	0.910	0.989	0.912	0.883	0.847	0.946	0.9^{6}	NA	0.811	0.920
IER2	5.8879	6.32×10^{-4}	0.995	0.975	0.607	0.882	0.979	0.929	0.932	NA	0.991	0.902
ARHGAP33	5.8763	6.45×10^{-4}	0.942	0.816	0.972	0.870	0.996	0.828	0.980	NA	0.778	0.957
ACOT7	5.8722	6.50×10^{-4}	0.635	0.898	0.963	0.996	0.788	0.999	0.936	NA	0.989	0.983
NEB	5.8591	6.65×10^{-4}	0.959	0.916	0.989	0.934	0.980	0.847	0.978	NA	0.850	0.697
KDR	5.8192	7.12×10^{-4}	0.879	0.792	0.990	0.689	0.960	0.963	0.961	NA	0.991	0.925
AJAP1	5.8168	7.15×10^{-4}	0.808	0.934	0.975	0.746	1.000	0.913	0.980	NA	0.894	0.879
PRKAR2B	5.8070	7.27×10^{-4}	0.858	0.995	0.897	0.929	0.806	0.815	0.915	NA	0.935	0.962
HMGCS1	5.7782	7.64×10^{-4}	0.856	0.992	0.886	0.960	0.840	0.913	0.937	NA	0.739	0.994
CLDN14	5.7747	7.69×10^{-4}	0.906	0.804	0.904	0.897	0.986	0.910	0.922	NA	0.958	0.814
INPP5K	5.7676	7.78×10^{-4}	0.580	0.941	0.969	0.967	0.871	0.988	0.969	NA	0.972	0.918
SSTR4	5.7653	7.81×10^{-4}	0.792	0.943	0.776	0.979	0.914	0.842	0.954	NA	0.965	0.946
EZR	5.7651	7.82×10^{-4}	0.951	0.767	0.961	0.831	0.981	0.916	0.864	NA	0.854	0.984
NR4A1	5.7445	8.10×10^{-4}	0.928	0.999	0.997	0.788	0.811	0.948	0.758	NA	0.985	0.905

FAM8A1	5.7301	8.30×10^{-4}	0.960	0.931	0.982	0.968	0.705	0.902	0.812	NA	0.995	0.862
COL11A1	5.7264	8.35×10^{-4}	0.664	0.899	0.917	0.919	0.963	0.980	0.977	NA	0.932	0.870
WDR19	5.7214	8.43×10^{-4}	0.830	0.943	0.993	0.990	0.709	0.922	0.997	NA	0.785	0.955
LAMP2	5.7205	8.44×10^{-4}	0.857	0.978	0.985	0.664	0.815	0.979	0.967	NA	0.969	0.916
CYLD	5.7079	8.63×10^{-4}	0.805	0.935	0.928	0.814	0.920	0.991	0.934	NA	0.917	0.842
IRF1	5.7050	8.67×10^{-4}	0.919	0.974	0.844	0.865	0.894	0.970	0.825	NA	0.979	0.817
SIGIRR	5.7000	8.75×10^{-4}	0.976	0.880	0.892	0.956	0.862	0.831	0.824	NA	0.991	0.873
NGLY1	5.6970	8.79×10^{-4}	0.997	0.878	0.989	0.969	0.685	0.865	0.829	NA	0.970	0.934
AVIL	5.6856	8.97×10^{-4}	0.758	0.919	0.839	0.946	0.788	0.998	0.976	NA	0.964	0.908
UBR2	5.6851	8.97×10^{-4}	0.974	0.657	0.955	0.941	0.900	0.998	0.859	NA	0.867	0.966
NFIL3	5.6837	8.99×10^{-4}	0.715	0.991	0.978	0.874	0.957	0.993	0.675	NA	0.987	0.970
ISG15	5.6815	9.03×10^{-4}	0.961	1.000	0.876	0.929	0.931	0.928	0.806	NA	0.862	0.790
RECK	5.6796	9.06×10^{-4}	0.957	0.814	0.690	0.880	0.980	0.960	0.948	NA	0.927	0.949
CYB5R1	5.6744	9.14×10^{-4}	0.982	0.940	0.944	0.932	0.566	0.966	0.996	NA	0.946	0.885
STX12	5.6720	9.18×10^{-4}	0.968	0.910	0.977	0.975	0.917	0.868	0.973	NA	0.985	0.579
TTC13	5.6715	9.19×10^{-4}	0.931	0.943	0.993	0.922	0.796	0.822	0.993	NA	0.993	0.714
L1TD1	5.6709	9.19×10^{-4}	0.943	0.881	0.972	0.871	0.938	0.738	0.926	NA	0.878	0.935
RRAD	5.6614	9.35×10^{-4}	0.884	0.958	0.982	0.948	0.699	0.787	0.966	NA	0.904	0.974
PIK3CA	5.6580	9.40×10^{-4}	0.870	0.952	0.932	0.760	0.986	0.919	0.905	NA	0.900	0.853
C7orf43	5.6551	9.45×10^{-4}	0.954	0.906	0.980	0.973	0.872	0.959	0.932	NA	0.891	0.646
ARC	5.6483	9.56×10^{-4}	0.973	0.943	0.878	0.972	0.603	0.860	0.989	NA	0.970	0.945
SNTG2	5.6465	9.59×10^{-4}	0.640	0.924	0.954	0.973	0.807	0.990	0.966	NA	0.897	0.967
FAM89B	5.6433	9.65×10^{-4}	0.987	0.916	0.914	0.966	0.676	0.838	0.982	NA	0.996	0.831

TLE4	5.6358	9.77×10^{-4}	0.872	0.764	0.872	0.994	0.912	0.966	0.967	NA	0.815	0.914
PER3	5.6345	9.79×10^{-4}	0.903	0.800	0.947	0.908	0.958	0.974	0.961	NA	0.990	0.665
PCMT1	5.6336	9.80×10^{-4}	0.996	1.000	0.993	0.966	0.760	0.875	0.623	NA	0.952	0.972
CHI3L1	5.6336	9.80×10^{-4}	0.947	0.899	0.930	0.961	0.757	0.904	0.910	NA	0.879	0.878
ATG4B	5.6311	9.85×10^{-4}	0.989	0.909	0.899	0.951	0.618	0.882	0.966	NA	0.973	0.931
BRD4	5.6309	9.85×10^{-4}	0.836	0.803	0.889	0.833	0.944	0.974	0.966	NA	0.852	0.972
IZUMO4	5.6247	9.96×10^{-4}	0.829	0.917	0.761	0.879	0.958	0.971	0.999	NA	0.870	0.889

Table S6. The recurrent relationships which significantly upregulated expression by BI–2536 treatment for 24 hours

Gene|ID	REC	FDR	Relative rank									
			A375	A549	HA1E	HCC515	HEPG2	HT29	MCF7	NPC	PC3	VCAP
IGF2R	13.7713	1.11×10^{-9}	0.996	0.993	0.996	0.988	NA	0.999	0.998	NA	0.987	0.976
INSIG1	12.3069	1.54×10^{-8}	0.997	0.964	0.995	0.982	NA	0.988	0.983	NA	0.998	0.992
HIST2H2BE	11.1286	1.25×10^{-7}	0.999	0.967	0.968	0.994	NA	0.983	0.999	NA	0.998	0.951
TIPARP	10.4700	4.21×10^{-7}	0.988	0.937	0.989	0.997	NA	0.978	0.958	NA	0.984	1.000
KIAA0226	10.4680	4.22×10^{-7}	0.990	0.961	0.989	0.966	NA	0.997	0.994	NA	0.979	0.953
ATP6V0D1	10.4679	4.22×10^{-7}	0.997	0.991	0.976	0.987	NA	0.995	0.980	NA	0.973	0.931
SLC25A4	10.3960	4.77×10^{-7}	0.998	0.995	0.957	0.923	NA	1.000	0.999	NA	0.993	0.963
HECA	10.2932	5.76×10^{-7}	0.970	0.953	0.982	0.996	NA	0.988	0.991	NA	0.981	0.959
STXBP1	10.1438	7.49×10^{-7}	0.994	0.934	0.971	0.996	NA	0.964	0.996	NA	0.974	0.986
HMGCR	10.1028	8.07×10^{-7}	1.000	0.973	0.988	0.994	NA	0.945	0.931	NA	0.988	0.992
RRAGC	9.9389	1.08×10^{-6}	0.991	0.994	0.994	0.945	NA	0.980	0.989	NA	0.924	0.986
TERF2IP	9.8642	1.23×10^{-6}	0.999	0.999	0.997	1.000	NA	0.829	0.999	NA	0.996	0.992
MAP1LC3B	9.7135	1.59×10^{-6}	0.976	0.931	0.956	0.992	NA	0.989	0.993	NA	0.993	0.959
CYTH2	9.6553	1.77×10^{-6}	0.978	1.000	0.988	0.974	NA	0.977	0.998	NA	0.886	0.989
NPC1	9.6164	1.89×10^{-6}	0.959	0.933	1.000	0.999	NA	0.968	0.989	NA	0.954	0.979
GDI1	9.1983	3.93×10^{-6}	0.990	0.918	0.999	0.956	NA	0.979	0.993	NA	0.995	0.927
NOSIP	9.1067	4.60×10^{-6}	0.948	0.992	0.996	0.956	NA	1.000	0.977	NA	0.890	0.993
CIRBP	9.0908	4.73×10^{-6}	0.971	0.945	0.997	0.923	NA	0.962	0.973	NA	0.994	0.981

LPIN1	8.9545	6.00×10^{-6}	0.970	0.920	0.991	0.983	NA	0.967	0.943	NA	0.972	0.992
HIST1H2AC	8.9521	6.03×10^{-6}	0.998	0.980	0.858	0.980	NA	0.972	0.994	NA	0.981	0.978
MGEA5	8.8075	7.78×10^{-6}	0.969	0.952	0.992	0.998	NA	0.995	0.982	NA	1.000	0.845
PLCG2	8.7748	8.24×10^{-6}	0.968	0.963	0.972	0.980	NA	0.965	0.964	NA	0.948	0.960
MAP3K3	8.7279	8.96×10^{-6}	0.954	0.860	0.998	0.996	NA	0.996	0.968	NA	0.952	1.000
WDR19	8.7037	9.35×10^{-6}	0.945	0.952	0.989	0.995	NA	0.963	0.993	NA	0.889	0.994
WDR47	8.6201	1.08×10^{-5}	0.996	0.984	0.932	0.949	NA	0.991	0.869	NA	0.995	0.997
POR	8.5963	1.13×10^{-5}	0.973	0.990	0.949	0.970	NA	0.956	0.989	NA	0.937	0.944
PI4K2A	8.4417	1.47×10^{-5}	0.980	0.825	0.988	0.991	NA	0.948	0.992	NA	0.981	0.999
H2BFS	8.4247	1.52×10^{-5}	0.997	0.959	0.857	0.964	NA	0.955	0.993	NA	0.980	0.993
HIST1H2BD	8.3869	1.62×10^{-5}	1.000	0.938	0.901	0.984	NA	0.975	0.991	NA	0.979	0.924
NXF1	8.3454	1.73×10^{-5}	0.927	0.963	0.966	0.950	NA	0.995	0.938	NA	0.995	0.952
EZR	8.2707	1.97×10^{-5}	0.972	0.962	0.972	0.955	NA	0.891	0.956	NA	0.988	0.983
FIG4	8.2261	2.13×10^{-5}	0.969	0.908	0.989	0.985	NA	0.952	0.892	NA	0.985	0.997
NTRK1	8.1747	2.33×10^{-5}	0.956	0.964	0.878	0.995	NA	0.922	0.987	NA	0.985	0.985
HIST1H2BK	8.1549	2.41×10^{-5}	0.997	0.947	0.916	0.959	NA	0.988	0.983	NA	0.976	0.904
HIST1H3H	8.1100	2.61×10^{-5}	0.999	0.898	0.856	0.984	NA	0.951	0.997	NA	0.990	0.996
DNAJC6	8.0968	2.66×10^{-5}	0.977	0.895	0.956	0.951	NA	0.965	0.984	NA	0.990	0.944
SPG7	8.0367	2.96×10^{-5}	0.998	0.889	0.982	0.982	NA	0.936	0.977	NA	0.958	0.934
ABCA5	7.9990	3.15×10^{-5}	0.920	0.940	0.982	0.994	NA	0.993	0.986	NA	0.988	0.854
RBM6	7.9398	3.48×10^{-5}	0.976	0.843	0.985	0.977	NA	0.995	1.000	NA	1.000	0.880
PPP2R5A	7.9225	3.59×10^{-5}	0.995	0.924	0.994	0.888	NA	0.997	0.883	NA	0.999	0.970
SERINC1	7.8676	3.94×10^{-5}	0.980	0.869	0.970	0.991	NA	0.985	0.985	NA	0.977	0.886

KDM3A	7.8263	4.23×10^{-5}	0.999	0.921	0.994	0.973	NA	0.975	0.834	NA	0.952	0.994
KIAA0513	7.7974	4.45×10^{-5}	0.978	0.838	0.975	0.990	NA	0.988	0.988	NA	0.998	0.886
ABCC10	7.7706	4.65×10^{-5}	0.979	0.967	0.989	0.872	NA	0.891	0.998	NA	0.972	0.965
FOXO3	7.7130	5.13×10^{-5}	0.924	0.806	0.999	0.989	NA	0.992	0.984	NA	0.942	0.999
PLEKHO2	7.6947	5.29×10^{-5}	0.823	0.989	0.998	0.992	NA	0.942	0.955	NA	0.948	0.981
CALCOCO1	7.6928	5.31×10^{-5}	0.990	0.882	0.970	0.952	NA	0.970	0.992	NA	0.873	0.995
AKR7A2	7.6793	5.43×10^{-5}	0.999	0.741	1.000	0.970	NA	0.994	0.991	NA	0.999	0.954
GLG1	7.5870	6.39×10^{-5}	0.978	0.998	0.944	0.921	NA	0.875	0.992	NA	0.960	0.943
AGTR2	7.5756	6.52×10^{-5}	0.988	0.984	0.868	0.946	NA	0.991	0.893	NA	0.981	0.960
BTG1	7.5275	7.10×10^{-5}	0.985	0.975	0.910	0.957	NA	0.899	0.957	NA	0.997	0.922
ENAH	7.5014	7.43×10^{-5}	0.975	0.981	0.963	0.856	NA	0.963	0.998	NA	0.945	0.919
ZCCHC14	7.4844	7.66×10^{-5}	0.994	0.985	0.993	0.988	NA	0.941	0.971	NA	0.869	0.862
IDI1	7.4831	7.67×10^{-5}	0.995	0.916	0.997	0.992	NA	0.922	0.857	NA	0.960	0.961
TPP1	7.4672	7.89×10^{-5}	0.965	0.948	0.934	0.943	NA	0.987	0.962	NA	0.957	0.897
KTN1	7.4588	8.02×10^{-5}	0.975	0.938	0.996	0.863	NA	0.973	0.996	NA	0.888	0.969
RCOR1	7.4444	8.21×10^{-5}	0.984	0.971	0.981	0.986	NA	0.994	0.995	NA	0.933	0.767
PLXNA3	7.4422	8.24×10^{-5}	0.939	0.936	0.986	0.964	NA	0.986	0.990	NA	0.970	0.828
TOB2	7.4086	8.75×10^{-5}	0.965	0.959	0.885	0.950	NA	0.986	0.897	NA	0.997	0.950
MTSS1	7.3927	8.98×10^{-5}	0.988	0.987	0.942	0.876	NA	0.909	0.978	NA	0.970	0.936
KLHL24	7.3293	1.00×10^{-4}	0.928	0.848	0.985	0.980	NA	0.958	0.938	NA	0.978	0.966
ARL8B	7.3125	1.03×10^{-4}	0.957	0.799	0.945	0.963	NA	0.990	0.973	NA	0.982	0.976
SC5D	7.3052	1.04×10^{-4}	0.957	0.968	0.957	0.993	NA	0.974	0.967	NA	0.939	0.825
HIST1H1C	7.2882	1.07×10^{-4}	0.998	0.955	0.864	0.917	NA	0.935	0.982	NA	0.999	0.925

RSRP1	7.2519	1.14×10^{-4}	0.988	0.927	0.981	0.922	NA	0.974	0.988	NA	0.993	0.806
CASP9	7.2484	1.15×10^{-4}	0.901	0.806	0.986	0.989	NA	0.932	0.999	NA	0.976	0.989
FAM117A	7.2414	1.16×10^{-4}	0.981	0.916	0.933	0.999	NA	0.995	0.955	NA	0.983	0.813
HIP1R	7.2165	1.21×10^{-4}	0.934	0.962	0.991	0.888	NA	0.898	0.988	NA	0.997	0.907
SQLE	7.2069	1.23×10^{-4}	0.982	0.939	0.965	0.977	NA	0.946	0.888	NA	0.916	0.948
TECPR2	7.1999	1.24×10^{-4}	0.937	0.999	0.959	0.896	NA	0.962	0.996	NA	0.972	0.846
JAK1	7.1803	1.28×10^{-4}	0.996	0.914	0.967	0.867	NA	0.943	0.959	NA	0.923	0.991
ABCA7	7.1642	1.32×10^{-4}	0.999	0.907	0.961	0.942	NA	0.987	0.981	NA	0.983	0.808
ZNF10	7.1562	1.34×10^{-4}	0.972	0.852	0.983	0.997	NA	0.978	0.961	NA	0.927	0.891
VCPIP1	7.1521	1.35×10^{-4}	0.875	0.967	0.984	0.970	NA	0.999	0.929	NA	0.894	0.938
DAZL	7.1521	1.35×10^{-4}	0.895	0.839	0.983	0.997	NA	0.990	0.973	NA	0.968	0.918
CSRNP2	7.1146	1.44×10^{-4}	0.960	0.718	0.987	0.981	NA	0.972	0.992	NA	0.983	0.990
PINK1	7.1056	1.46×10^{-4}	0.995	0.863	0.964	0.997	NA	0.976	0.999	NA	0.937	0.829
OGDH	7.0967	1.48×10^{-4}	0.963	0.959	0.966	0.962	NA	0.897	0.998	NA	0.928	0.876
AKAP13	7.0885	1.50×10^{-4}	0.977	0.945	0.924	0.932	NA	0.957	0.979	NA	0.858	0.974
ACTR1A	7.0739	1.54×10^{-4}	0.995	0.779	0.965	0.991	NA	0.969	0.981	NA	0.930	0.949
RYBP	7.0349	1.65×10^{-4}	0.980	0.791	0.934	0.963	NA	0.999	0.986	NA	0.981	0.918
MYO5A	6.9725	1.84×10^{-4}	0.973	0.866	0.863	0.969	NA	0.963	0.955	NA	0.958	0.987
OSBP	6.9558	1.89×10^{-4}	0.921	0.851	0.965	0.964	NA	0.951	0.990	NA	0.978	0.910
LRPAP1	6.9544	1.89×10^{-4}	0.996	0.991	1.000	0.991	NA	1.000	1.000	NA	0.988	0.633
SQSTM1	6.9465	1.92×10^{-4}	0.946	0.940	0.967	0.875	NA	0.902	0.953	NA	0.959	0.983
CDKN2D	6.9405	1.94×10^{-4}	0.935	0.963	0.955	0.928	NA	0.986	0.995	NA	0.941	0.827
GTF2B	6.9359	1.95×10^{-4}	0.792	0.923	0.989	0.955	NA	0.996	0.990	NA	0.925	0.968

IER5	6.9308	1.97×10^{-4}	0.968	0.966	0.995	0.870	NA	0.908	0.929	NA	0.955	0.934
BTG2	6.8942	2.09×10^{-4}	0.874	0.966	0.951	0.988	NA	0.846	0.956	NA	0.958	0.985
TSC2	6.8819	2.14×10^{-4}	0.907	0.846	0.963	0.956	NA	0.967	0.950	NA	0.953	0.977
SUGP1	6.8356	2.31×10^{-4}	0.929	0.998	0.999	0.975	NA	0.860	0.977	NA	0.796	0.995
IZUMO4	6.8202	2.37×10^{-4}	0.988	0.976	0.954	0.968	NA	0.998	0.995	NA	0.896	0.755
PARD6B	6.8155	2.39×10^{-4}	0.967	1.000	0.985	0.964	NA	0.986	0.702	NA	0.962	0.978
CRK	6.8109	2.41×10^{-4}	0.959	0.788	0.993	0.972	NA	0.982	0.899	NA	0.937	0.990
CAP1	6.7961	2.47×10^{-4}	0.958	0.993	0.937	0.757	NA	0.979	0.992	NA	0.940	0.968
YPEL5	6.7867	2.51×10^{-4}	0.982	0.856	0.836	0.988	NA	0.913	0.977	NA	0.966	0.994
RIPK1	6.7405	2.72×10^{-4}	0.983	0.772	0.932	0.979	NA	0.969	0.965	NA	0.960	0.951
TMEM50A	6.7372	2.73×10^{-4}	0.997	0.674	1.000	0.968	NA	1.000	0.999	NA	0.996	0.912
NEU1	6.7350	2.75×10^{-4}	0.993	0.920	0.993	0.977	NA	0.930	0.984	NA	0.847	0.859
KLHDC2	6.7338	2.75×10^{-4}	0.997	0.674	0.985	0.973	NA	1.000	0.947	NA	0.977	0.992
HAGH	6.7209	2.81×10^{-4}	0.992	0.942	0.999	0.814	NA	0.998	0.984	NA	0.864	0.915
AP3D1	6.7150	2.84×10^{-4}	0.954	0.967	0.968	0.789	NA	0.955	0.974	NA	0.951	0.944
ULK1	6.7135	2.85×10^{-4}	0.975	0.819	0.964	0.986	NA	0.983	0.999	NA	0.790	1.000
FDFT1	6.6677	3.08×10^{-4}	0.966	0.946	0.974	0.834	NA	0.993	0.860	NA	0.998	0.923
STX12	6.6525	3.16×10^{-4}	0.957	0.689	0.971	0.972	NA	0.970	0.997	NA	0.991	0.977
HIST1H2BH	6.6243	3.31×10^{-4}	0.999	0.950	0.714	0.928	NA	0.950	0.991	NA	0.992	0.988
CCDC92	6.5972	3.46×10^{-4}	0.928	0.813	0.939	0.994	NA	0.996	0.989	NA	0.997	0.835
OR11A1	6.5819	3.56×10^{-4}	0.800	0.985	0.938	0.995	NA	0.924	0.902	NA	0.986	0.952
GTF3C1	6.5727	3.61×10^{-4}	0.964	0.999	0.991	0.947	NA	0.835	0.991	NA	0.854	0.901
NAGK	6.5653	3.65×10^{-4}	0.982	0.894	0.974	0.966	NA	0.973	0.973	NA	0.861	0.852

FBXO42	6.5575	3.70×10^{-4}	0.979	0.955	0.896	0.993	NA	0.938	0.916	NA	0.895	0.895
SERINC5	6.5359	3.84×10^{-4}	0.966	0.969	0.877	0.944	NA	0.965	0.979	NA	0.966	0.808
MYCBP2	6.5216	3.93×10^{-4}	0.981	0.998	0.998	0.763	NA	0.910	1.000	NA	0.988	0.850
CALCR	6.5137	3.99×10^{-4}	0.879	0.906	0.941	0.955	NA	0.933	0.929	NA	0.983	0.934
ATXN2	6.5017	4.07×10^{-4}	0.977	0.802	0.949	0.947	NA	0.939	0.971	NA	0.947	0.934
MAST3	6.4865	4.18×10^{-4}	0.977	0.930	0.953	0.770	NA	0.990	0.978	NA	0.968	0.906
KCTD13	6.4750	4.26×10^{-4}	0.968	0.911	0.942	0.867	NA	0.999	0.898	NA	0.937	0.935
SPSB3	6.4675	4.31×10^{-4}	0.984	0.873	0.980	0.898	NA	0.818	0.932	NA	0.985	0.996
SREBF2	6.4641	4.33×10^{-4}	0.997	0.983	0.982	0.937	NA	0.850	0.810	NA	0.973	0.933
ZPBP	6.4526	4.42×10^{-4}	0.939	0.949	0.886	0.925	NA	0.927	0.968	NA	0.894	0.961
CDKN1A	6.4099	4.76×10^{-4}	0.999	0.989	0.979	0.885	NA	0.957	0.851	NA	0.993	0.808
LSS	6.3819	4.98×10^{-4}	0.982	0.942	0.980	0.986	NA	0.805	0.971	NA	0.963	0.827
HERC1	6.3223	5.51×10^{-4}	0.964	0.854	0.967	0.943	NA	0.969	0.953	NA	0.980	0.810
ANKRD11	6.2930	5.78×10^{-4}	0.950	0.954	0.929	0.887	NA	0.966	0.940	NA	0.934	0.866
MARK3	6.2911	5.80×10^{-4}	0.933	0.953	0.830	0.957	NA	0.974	0.992	NA	0.995	0.807
FDPS	6.2839	5.87×10^{-4}	0.974	0.947	0.972	0.976	NA	0.949	0.913	NA	0.867	0.831
CDIPT	6.2669	6.04×10^{-4}	0.947	0.971	0.978	0.895	NA	0.986	0.986	NA	0.972	0.716
FAM49A	6.2656	6.05×10^{-4}	0.929	0.857	0.944	0.926	NA	0.892	0.921	NA	0.994	0.958
RHCE	6.2584	6.13×10^{-4}	0.884	0.850	0.936	0.963	NA	0.918	0.995	NA	0.950	0.925
IP6K2	6.2370	6.35×10^{-4}	0.950	0.844	0.922	0.999	NA	0.951	0.968	NA	0.986	0.808
NRBF2	6.2321	6.40×10^{-4}	0.821	0.943	0.938	0.995	NA	0.980	0.885	NA	0.960	0.900
USP9X	6.2266	6.46×10^{-4}	0.983	0.742	0.991	0.989	NA	0.985	0.976	NA	0.925	0.851
PLXNB3	6.2054	6.68×10^{-4}	0.958	0.994	0.916	0.786	NA	0.930	0.985	NA	0.907	0.945

C18orf8	6.1917	6.83×10^{-4}	0.907	0.703	0.982	0.933	NA	0.974	0.976	NA	0.968	0.999
BRD2	6.1910	6.84×10^{-4}	0.935	0.957	0.909	0.974	NA	0.995	0.978	NA	0.922	0.755
MSMO1	6.1654	7.14×10^{-4}	0.995	0.936	0.987	0.983	NA	0.829	0.862	NA	0.852	0.970
SLC31A2	6.1414	7.44×10^{-4}	0.880	0.996	0.943	0.940	NA	0.869	0.969	NA	0.912	0.892
UBN1	6.1200	7.71×10^{-4}	0.829	0.880	0.978	0.903	NA	0.894	0.972	NA	0.985	0.961
DKFZP586I1420	6.1097	7.84×10^{-4}	0.935	0.942	0.962	0.941	NA	0.915	0.889	NA	0.862	0.945
NXPH4	6.1038	7.92×10^{-4}	0.930	0.885	0.870	0.912	NA	0.972	0.882	NA	0.950	0.993
ABCD1	6.0986	7.99×10^{-4}	0.879	0.997	0.960	0.843	NA	0.984	0.858	NA	0.934	0.943
PPIEL	6.0850	8.18×10^{-4}	0.761	0.962	0.872	0.998	NA	0.942	0.964	NA	0.976	0.931
AKIRIN1	6.0846	8.18×10^{-4}	0.985	0.920	0.977	0.856	NA	0.940	0.997	NA	0.890	0.831
GADD45A	6.0728	8.34×10^{-4}	0.949	0.919	0.689	0.964	NA	0.935	0.996	NA	0.997	0.974
PIAS1	6.0612	8.50×10^{-4}	0.983	0.637	0.915	0.993	NA	0.984	0.999	NA	0.995	0.940
SOX3	6.0452	8.73×10^{-4}	0.969	0.998	0.957	0.960	NA	0.995	0.673	NA	0.891	0.984
DUSP14	6.0321	8.93×10^{-4}	0.986	0.920	0.955	0.969	NA	0.997	0.959	NA	0.775	0.835
BMPR2	6.0243	9.04×10^{-4}	0.977	0.979	0.954	0.709	NA	0.965	0.971	NA	0.879	0.975
RB1CC1	6.0216	9.08×10^{-4}	0.896	0.847	0.935	0.904	NA	0.988	0.931	NA	0.943	0.933
ATP6V0A1	5.9755	9.78×10^{-4}	0.982	0.908	0.988	0.931	NA	0.996	0.937	NA	0.961	0.699
NCALD	5.9728	9.83×10^{-4}	0.876	0.888	0.930	0.936	NA	0.983	0.989	NA	0.957	0.814

Table S7. All sequences of primer used for qRT–PCR

Gene	Forward Primer	Reverse Primer
MCM2	GCC AAG ATG TAC AGT GAC CTG A	GAT GTG CCG CAC CGT AAT
MCM10	GCA TGA TGG TGT GAA GAG GTT	TCC CAT TTG TAG AGG CCA CAG
GAP43	TCC GTG GAC ACA TAA CAA GG	CAG TAG TGG TGC CTT CTC C
CRT	AAG TTC TAC GGT GAC GAG GAG	GTC GAT GTT CTG CTC ATG TTT C
NSE2	TGT CTG CTG CTC AAG GTC AA	CGA TGA CTC ACC ATG ACC C
NEFH	TGA ACA CAG ACG CTA TGC GCT CAG	CAC CTT TAT GTG AGT GGA CAC AGA G
GAPDH	ACA CCC ACT CCT CCA CCT TTG	GCT GTA GCC AAA TTC GTT GTC ATA C
